# Dynamic heterogeneity of colorectal cancer during progression revealed clinical risk-associated cell types and regulations in single-cell resolution and spatial context

**DOI:** 10.1093/gastro/goad034

**Published:** 2023-06-24

**Authors:** Haoxian Ke, Zhihao Li, Peisi Li, Shubiao Ye, Junfeng Huang, Tuo Hu, Chi Zhang, Ming Yuan, Yuan Chen, Xianrui Wu, Ping Lan

**Affiliations:** Department of General Surgery (Colorectal Surgery), The Sixth Affiliated Hospital, Sun Yat-sen University, Guangzhou, Guangdong, P. R. China; Guangdong Provincial Key Laboratory of Colorectal and Pelvic Floor Diseases, Guangdong Institute of Gastroenterology ,The Sixth Affiliated Hospital, Sun Yat-sen University, Guangzhou, Guangdong, P. R. China; Department of General Surgery (Colorectal Surgery), The Sixth Affiliated Hospital, Sun Yat-sen University, Guangzhou, Guangdong, P. R. China; Guangdong Provincial Key Laboratory of Colorectal and Pelvic Floor Diseases, Guangdong Institute of Gastroenterology ,The Sixth Affiliated Hospital, Sun Yat-sen University, Guangzhou, Guangdong, P. R. China; Guangdong Provincial Key Laboratory of Colorectal and Pelvic Floor Diseases, Guangdong Institute of Gastroenterology ,The Sixth Affiliated Hospital, Sun Yat-sen University, Guangzhou, Guangdong, P. R. China; School of Medicine, Sun Yat-sen University, Shenzhen, Guangdong, P. R. China; Guangdong Provincial Key Laboratory of Colorectal and Pelvic Floor Diseases, Guangdong Institute of Gastroenterology ,The Sixth Affiliated Hospital, Sun Yat-sen University, Guangzhou, Guangdong, P. R. China; Department of General Surgery (Colorectal Surgery), The Sixth Affiliated Hospital, Sun Yat-sen University, Guangzhou, Guangdong, P. R. China; Guangdong Provincial Key Laboratory of Colorectal and Pelvic Floor Diseases, Guangdong Institute of Gastroenterology ,The Sixth Affiliated Hospital, Sun Yat-sen University, Guangzhou, Guangdong, P. R. China; Department of General Surgery (Colorectal Surgery), The Sixth Affiliated Hospital, Sun Yat-sen University, Guangzhou, Guangdong, P. R. China; Guangdong Provincial Key Laboratory of Colorectal and Pelvic Floor Diseases, Guangdong Institute of Gastroenterology ,The Sixth Affiliated Hospital, Sun Yat-sen University, Guangzhou, Guangdong, P. R. China; Department of General Surgery (Colorectal Surgery), The Sixth Affiliated Hospital, Sun Yat-sen University, Guangzhou, Guangdong, P. R. China; Guangdong Provincial Key Laboratory of Colorectal and Pelvic Floor Diseases, Guangdong Institute of Gastroenterology ,The Sixth Affiliated Hospital, Sun Yat-sen University, Guangzhou, Guangdong, P. R. China; Department of General Surgery (Colorectal Surgery), The Sixth Affiliated Hospital, Sun Yat-sen University, Guangzhou, Guangdong, P. R. China; Guangdong Provincial Key Laboratory of Colorectal and Pelvic Floor Diseases, Guangdong Institute of Gastroenterology ,The Sixth Affiliated Hospital, Sun Yat-sen University, Guangzhou, Guangdong, P. R. China; School of Medicine, Sun Yat-sen University, Shenzhen, Guangdong, P. R. China; Department of General Surgery (Colorectal Surgery), The Sixth Affiliated Hospital, Sun Yat-sen University, Guangzhou, Guangdong, P. R. China; Guangdong Provincial Key Laboratory of Colorectal and Pelvic Floor Diseases, Guangdong Institute of Gastroenterology ,The Sixth Affiliated Hospital, Sun Yat-sen University, Guangzhou, Guangdong, P. R. China; Department of General Surgery (Colorectal Surgery), The Sixth Affiliated Hospital, Sun Yat-sen University, Guangzhou, Guangdong, P. R. China; Guangdong Provincial Key Laboratory of Colorectal and Pelvic Floor Diseases, Guangdong Institute of Gastroenterology ,The Sixth Affiliated Hospital, Sun Yat-sen University, Guangzhou, Guangdong, P. R. China

**Keywords:** colorectal cancer, tumor heterogeneity, tumor progression, single-cell RNA sequencing, spatial transcription sequencing

## Abstract

**Background:**

Tumor heterogeneity is contributed by tumor cells and the microenvironment. Dynamics of tumor heterogeneity during colorectal cancer (CRC) progression have not been elucidated.

**Methods:**

Eight single-cell RNA sequencing (scRNA-seq) data sets of CRC were included. Milo was utilized to reveal the differential abundance of cell clusters during progression. The differentiation trajectory was imputed by using the Palantir algorithm and metabolic states were assessed by using scMetabolism. Three spatial transcription sequencing (ST-seq) data sets of CRC were used to validate cell-type abundances and colocalization. Cancer-associated regulatory hubs were defined as communication networks affecting tumor biological behaviors. Finally, quantitative reverse transcription polymerase chain reaction and immunohistochemistry staining were performed for validation.

**Results:**

TM4SF1^+^, SOX4^+^, and MKI67^+^ tumor cells; CXCL12^+^ cancer-associated fibroblasts; CD4^+^ resident memory T cells; Treg; IgA^+^ plasma cells; and several myeloid subsets were enriched in stage IV CRC, most of which were associated with overall survival of patients. Trajectory analysis indicated that tumor cells from patients with advanced-stage CRC were less differentiated, when metabolic heterogeneity showed a highest metabolic signature in terminal states of stromal cells, T cells, and myeloid cells. Moreover, ST-seq validated cell-type abundance in a spatial context and also revealed the correlation of immune infiltration between tertiary lymphoid structures and tumors followed by validation in our cohort. Importantly, analysis of cancer-associated regulatory hubs revealed a cascade of activated pathways including leukocyte apoptotic process, MAPK pathway, myeloid leukocyte differentiation, and angiogenesis during CRC progression.

**Conclusions:**

Tumor heterogeneity was dynamic during progression, with the enrichment of immunosuppressive Treg, myeloid cells, and fibrotic cells. The differential state of tumor cells was associated with cancer staging. Assessment of cancer-associated regulatory hubs suggested impaired antitumor immunity and increased metastatic ability during CRC progression.

## Introduction

Colorectal cancer (CRC) is one of the most common malignancies and leading causes of cancer-related death worldwide [1, 2]. Although the mortality of CRC is reduced thanks to cancer screening and early detection in developed countries, it upsurges in developing countries [3, 4]. CRC can be often ranged into stages from I to IV, when early stages (I and II) indicate local infiltration and advanced stages (III and IV) refer to cancer dissemination to lymph nodes or distant organs. Surgical resection is the primary therapeutic strategy for early-stage CRC, while a combination of therapeutic regimens that includes but is not limited to chemotherapy, targeted therapy, and immunotherapy are used to improve prognosis for patients with advanced CRC. The 5-year survival rate for stage I–II CRC was 89.9%, while it dropped to 14.2% for stage IV CRC [1]. Since therapeutic strategies are limited in improving outcomes, it is important to find novel therapeutic targets for patients with advanced CRC.

High heterogeneity was observed in CRC, contributed by tumor cells and their tumor microenvironment (TME). Numerous studies have focused on the molecular mechanisms of increased heterogeneity in CRC, such as genetic alteration, transcriptome, non-coding RNA regulation, cancer-associated protein, and metabolism [5–9]. Three different pathways of genomic instability, including chromosomal instability, microsatellite instability, and CpG island methylation, have been recognized in the complex development of CRC. As for transcription, single-cell RNA sequencing (scRNA-seq) unveiled increased heterogeneity during the development of CRC from adenoma [10]. Moreover, consensus molecular subtypes (CMS) based on RNA-seq identified four subtypes for CRC, including CMS1 (immune), CMS2 (canonical), CMS3 (metabolic), and CMS4 (mesenchymal) [11]. These subtypes took TME into account, suggesting the existence of intrinsic features of tumor heterogeneity. TME had a dynamic composition of stromal cells, immune cells, and extracellular factors that surrounded cancer cells. Immune checkpoints such as PD-1 and CTLA-4 expressed in immune cell have been discovered to be novel therapeutic targets, suggesting the important role of TME in antitumor immunity [12, 13]. However, only a small fraction of patients with microsatellite instability-high CRC are suitable for immunotherapy. A better understanding of regulatory hubs in the progression of CRC might help reveal new therapeutic targets. On the other hand, metabolic reprogramming occurs not only in tumor cells but also in stromal and immune cells [9, 14]. Heterogeneity of tumor metabolism determines molecular features as well as prognosis. Furthermore, the metabolic crosstalk between the tumor cells and factors of the TME facilitate tumor progression, metastasis, and immune escape. Taken together, some characteristics of tumors and TME were demonstrated by genomics, transcriptomics, and proteomics, whereas intrinsic features of CRC heterogeneity were still not elaborated.

Although analysis of different transcriptomic features between tumor and normal mucosa is essential and is able to unveil potential therapeutic targets and early screening markers, the difference in genomic, transcriptomic, and proteomic features between early-stage and advanced-stage tumors is also important but has been rarely reported [15–17]. Several studies assessed the altered features of CRC during progression based on genomic or transcriptomic sequencing [5, 18–20]. Limited by a mixture of RNA-seq, the source of heterogeneity was difficult to be identified. Recently scRNA-seq has been used to explicate some intrinsic characteristics of several cancer types and elaborated the source of tumor heterogeneity, showing the ability to deconvolute cellular complexity and cell–cell interaction in tumors [21–23]. For example, by performing scRNA-seq on cohorts of Samsung medical center and Katholieke Universiteit Leuven, the heterogeneity of TME was unveiled for CRC and two intrinsic malignant epithelial cell types were recognized by combing more scRNA-seq data afterwards [24, 25]. On the other hand, spatial transcription sequencing (ST-seq) can indicate the spatial context of various cell types, making it a powerful method to study tumor heterogeneity [26]. The landscape and dynamics of CRC that evolves from early stage to advanced stage have not been elucidated at the single-cell level when considering the spatial context.

Here, we comprehensively studied the dynamic features of tumors and stromal and immune cells in stage I–IV CRC to reveal clinical risk–associated cell types and regulations by collecting public bulk RNA-seq, scRNA-seq, and ST-seq data sets, and validating results by using an independent cohort. The abundance of epithelial, stromal, and immune cells was found to be altered during tumor progression, suggesting that TME underwent remodeling. TM4SF1^+^ malignant epithelial cells were enriched in stage IV CRC. We also observed the trajectory of tumor cells and found that tumor cells with more differentiated potential were enriched in advanced CRC. Furthermore, the abundance, functions, and lineages of stromal and immune cells in TME were also demonstrated, making it clearer regarding their landscape and dynamics during CRC progression. Finally, intercellular communication networks were inquired to unveil cancer-associated regulatory hubs in different stages, showing the cascade of activated pathways related to regulation of antitumor immunity, tumor progression, as well as the ability of metastasis. LIF–LIFR was identified to be an important cancer-associated regulatory hub in advanced CRC and patients with higher expression of *LIF* in CRC tissue were associated with a lower overall survival (OS) rate.

## Materials and methods

### Patient and tissue sample collection

Tissue from tumors as well as invasive margins of 80 patients with CRC who were operated on at the Sixth Affiliated Hospital of Sun Yat-sen University was collected. Clinical information of these patients is provided in Supplementary Table 1. This study was approved by the Ethical Committee of the Sixth Affiliated Hospital of Sun Yat-sen University (Approval No. G2020001). Written informed consent was provided by all patients.

### Collection of scRNA-seq and ST-seq data sets

Data sets on scRNA-seq from the Gene Expression Omnibus (GEO) database were included if they met the following criteria: (i) using 10x Genomics scRNA-seq, (ii) evaluating CRC tissues from January 2019 to June 2022, and (iii) having available CRC stage information. A total of eight data sets were included (Supplementary Table 2).

As for ST-seq data sets, to minimize the discrepancies and batch effect across sequencing platforms, only ST-seq data sets generated from the 10x Genomics Visium platform were enrolled for analysis. Besides, data sets without hematoxylin and eosin (H & E) staining images were excluded. Finally, three data sets including one stage II CRC sample, two stage IV CRC samples, and four CRC border samples without stage information were enrolled for further analysis (Supplementary Table 3).

### Analysis of scRNA-seq data

Seurat workflow (version 4.1.0) was used to analyse scRNA-seq data. Cells with >250 genes, >1,000 unique molecular identifiers (UMIs), and <20% mitochondrial gene expression in UMI counts were selected for further analysis. Python package scrublet (version 0.2.3) was used to remove doublets for each sample. Then, counts data were normalized with pseudo-count 10,000 and followed by log-transformation using an offset of 1, and gene expression was also scaled. Next, FindVariableFeatures was used to get 2,000 of the most variant genes, followed by principal component analysis. Harmony algorithm was used to correct batch effect. Then, ElbowPlot function was used to determine the number of corrected principal components being used for clustering and Uniform Manifold Approximation and Projection (UMAP). Subsequently, cell clusters were identified by using FindNeighbors and FindCluster function, and resolutions from 0.1 to 1.2 were explored for best clustering.

### Differential abundance analysis of cell types by scRNA-seq data

Milo (version 1.2.0) was used to test for differential abundance among samples from stages I to IV. We constructed a *k*-nearest neighbor graph and assigned cells to neighborhoods. Then, we calculated distance and counted the number of cells belonging to each sample in each neighborhood. Each neighborhood was assigned a cell-type label based on majority voting of cells in this neighborhood. A “mixed” label would be assigned if the number of the most abundant label was <70% of cells and this neighborhood would be removed. To test the differential abundance across stages, we divided samples into stage I–IV groups and the cell count of neighborhoods was modeled using a negative binomial generalized linear model. Multiple testing was controlled by using the weighted Bonferroni–Hochberg procedure correction. If the number of a specific cluster in groups was not enough for statistics, this cluster would be removed from differential abundance analysis.

### Differentiation trajectory analysis

Palantir algorithm was used to align cells along differentiation trajectories. Briefly, diffusion maps were constructed and the low dimensional embedding of data was estimated based on the eigen-gap. Next, MAGIC was used to impute data for visualization and determining gene expression trends. Then, an annotated cell was identified as early cell and Palantir was run to determine differentiation trajectories.

A single-cell trajectory was also analysed by using monocle3 (version 1.0.0). After clustering and dimensionality reduction, cells were partitioned into trajectories followed by learning the principal graph. The naive state of lineages was recognized as the root node.

The CytoTRACE algorithm was used to validate the differentiation of malignant epithelial cells. First, counts of malignant epithelial cells were normalized with a pseudo-count of 10,000 followed by log_2_ transformation using an offset of 1. Batch effects were corrected by matching mutual nearest neighbors. Then the Pearson correlation between each gene’s normalized expression and gene counts was calculated and the geometric mean expression of the top 200 genes most positively correlated with gene counts was defined as the gene counts signature, which was used to run the CytoTRACE procedure. The output value of each cell was ranked and scaled between 0 and 1, suggesting their relative differentiation status. Zero represents more differentiated while 1 represents less differentiated.

### Characterizing metabolism from scRNA-seq data

Metabolic states were analysed by using the scMetabolism package (version 0.2.1), which applied the VISION algorithm to calculate the activity score of each cell in 80 metabolic pathways with default parameters. To overcome the sparsity of the scRNA-seq data, MAGIC imputed data were used for scMetabolism. We also compared metabolic scores of each cluster using a Wilcoxon rank-sum test with the Bonferroni**–**Hochberg procedure.

### Calculating the trend of gene expression or metabolic activity for branching trajectory

R package gam was used to apply a generalized additive model to predict the trend of gene expression or metabolic activity during differentiation. To begin with, cells with a branch probability calculated by using Palantir of <0.7 were removed. Then, the model was fit for pseudo-time and values of expression. Probability was used as the weight in the fitting process. Finally, the predicted values of 500 bins along the pseudo-time were returned and their standard deviations were also calculated for plotting.

### Analysis of the copy-number variation

InferCNV (version 1.10.1) was used to identify evidence for large-scale chromosomal copy-number variations from a single tumor cell. Normal epithelial cells from GSE132465 were applied as the reference and parameters were set to default values.

### Identification of intercellular communications in CRC

Intercellular communication networks were analysed by using CellChat (version 1.5.0) by evaluating the expression of paired ligands and receptors within cell populations. A cell–cell communication network was inferred by assigning each interaction with a probability value and a permutation test was performed.

### Calculate module scores of pathway signaling for scRNA-seq data

Three hallmark gene sets were downloaded from the Molecular Signatures Database (http://www.gsea-msigdb.org/gsea/msigdb) for analysis: (i) gene sets of epithelial–mesenchymal transition (EMT), HALLMARK_EPITHELIAL_MESENCHYMAL_TRANSITION; (ii) gene sets of T-cell receptor (TCR) signaling, KEGG_T_CELL_RECEPTOR_SIGNALING_PATHWAY; (iii) gene sets of B-cell receptor (BCR) signaling, KEGG_B_CELL_RECEPTOR_SIGNALING_PATHWAY. Gene sets used to calculate T-cell cytotoxic scores included *CST7*, *GZMA*, *GZMB*, *IFNG*, *NKG7*, and *PRF1*, when five exhaustion marker genes (*CTLA4*, *HAVCR2*, *LAG3*, *PDCD1*, and *TIGIT*) were used to calculate T-cell exhaustion scores, which were reported previously [24]. The AddModuleScore function of the Seurat package was used to calculate the expression levels of selected gene sets with default parameters while MAGIC imputed data were utilized.

### The regulon activity of transcription factors using SCENIC

The Python version of SCENIC algorithm pySCENIC (version 0.12.1) was used to assess the regulatory networks in individual cells. A motif data set was utilized to construct regulons for each transcription factor and the co-expressed genes for each transcription factor were computed by using GENIE3. Then Spearman’s correlation between transcription factors and potential targets was calculated. Finally, regulon activity was analysed by using AUCell.

### Gene ontology enrichment analysis

Gene ontology (GO) enrichment analysis was performed using the R package clusterProfiler (version 4.2.2). The results of marker gene identification for cell types using the Seurat package FindAllMarkers function, epithelium-associated genes in gene clusters, and enriched ligands or receptors expressed in a specific CRC stage were input.

### Analysis of ST-seq data

Spots in Visium slices with >500 genes and <30% mitochondrial gene expression in UMI counts were selected for the following analysis. Normalizing spots and finding variable features were processed by using the SCTransform function with default parameters. Next, principal component analysis and clustering were performed while the optimal number of principal components selected for finding neighborhoods was determined by using the ElbowPlot function, and resolutions ranging from 0.1 to 1.2 were explored for best clustering.

### Colocalization analysis of ST-seq data

Cell2location (version 0.1) was applied to estimate the cell abundance of each spot. Briefly, to train the reference model, we removed lowly expressed genes and cell types that consisted of <30 cells in each stage, followed by sampling a maximum of 1,000 cells for each cell type. Then spatial cell-type deconvolution was performed using default parameters. To identify the microenvironments of co-localizing cell types, we applied nonnegative matrix factorization to the matrix of estimated abundance, which was factorized into matrices W and H. H matrix was used to assign spots with the latent factor that had the largest rank value scaled by its mean, while W matrix represented the weight of each cell type contributing to the latent factor. Here latent factors were defined as a set of colocalized cell types that were made up of the tissue microenvironment as reported previously [27]. The number of latent factors was determined by using the complexity of tissue morphology. Factors ranging from 9 to 13 were tested and final clustering of spots defined by 11 factors was shown to be similar to the tissue morphology.

### Analysis of the bulk RNA sequencing data from The Cancer Genome Atlas or GEO

The counts matrix generated by using the STAR analysis pipeline and clinical data only with tumor samples from The Cancer Genome Atlas (TCGA)-colon adenocarcinoma (COAD) cohort (*n *=* *456) and TCGA**-**rectum adenocarcinoma (READ) cohort (*n *=* *166) were acquired using the TCGAbiolinks package (version 2.24.3). Counts data were normalized to counts per million followed by log2 transformation using an offset of 1. A list of RNA-seq data from GEO that was reported previously [28] were collected. Then, data sets with available survival data were selected for analysis.

Survminer (version 0.4.9) and survival (version 3.4.0) packages were used for survival analysis. Potential cutting points were repeatedly tested to find the maximum rank statistic, followed by applying to perform the dichotomy of cell fraction or gene expression, which divided patients into two groups. The two-sided long-rank test was performed for comparison of Kaplan–Meier survival curves.

### Impute cell fractions for bulk RNA-seq data

Single-cell expression matrix was uploaded to CIBERSORTx online analysis platform to infer cell-type-specific gene expression profiles according to the instructions. Then mixture data sets from TCGA**–**COAD, TCGA**–**READ, GSE17536, GSE17537, and GSE39582 were deconvoluted. The relative proportions of cell types were obtained for each sample. To validate the results of CIBERSORTx, we also estimated cell-type-specific enrichment scores for samples from TCGA**–**COAD by using the ConsensusTME package (version 0.0.1.9000), which had generated cancer-specific signatures for multiple cell types in TME. The gene set for COAD was selected to run the gene set variation analysis (GSVA) algorithm.

### Assessment of Klintrup–Mäkinen score

The images of H & E staining of the tumor or invasive margin were utilized to estimate immune infiltration as previously reported [29]. Briefly, a score of 0 indicated absence of an immune reaction and 1 indicated a weak, 2 indicated a moderate, and 3 indicated a severe increase in immune cells.

### RNA extraction and qRT–PCR

RNA was extracted from CRC tissues by using TRIzol Reagent (15596026; Invitrogen, Carlsbad, CA, USA) followed by quantification using a NanoDrop^TM^ ND-2000 spectrophotometer. Next, the ReverTra Ace qPCR RT Kit (FSQ-101; TOYOBO, Osaka, Japan) was used to perform reverse transcription following the manufacturer’s instructions. We conducted quantitative reverse transcription polymerase chain reaction (qRT–PCR) in the Applied Biosystems 7500 Sequence Detection system.

### Immunohistochemistry

Paraffin-embedded sections were routinely dewaxed and hydrated, followed by antigen retrieval using Tris/EDTA pH 9.0 buffer. Then slices were incubated with 3% hydrogen peroxide to inactivate endogenous peroxidase for 10 min. After being washed using PBS three times, slices were blocked in normal goat serum for 1 h and incubated with rabbit anti-LIF (26757–1-AP; Proteintech, Rosemont, IL, USA) at 4°C overnight. After being washed using TBST three times, slices were further incubated with horseradish peroxidase conjugated anti-rabbit IgG (DS-0003; Zhongshan Gold Bridge Biological Technology, Guangdong, China) at room temperature for 1 h followed by washing. Diaminobenzidine (ZLI-9017; Zhongshan Gold Bridge Biological Technology) was used for enzymatic detection. Finally, slices were counterstained, dehydrated, cleared, and mounted.

### Statistical analysis

R (http://www.r-project.org) was used for statistical analysis and graphing. ANOVA was used to determine whether there were any statistically significant differences between the mean values of more than two groups when Tukey’s test was performed for multiple comparisons. The *P*-value for Pearson’s correlation coefficients was calculated using a *t*-distribution with n-2 degrees of freedom that was performed using R package ggpmisc. It was considered as statistically significance when *P* was <0.05.

## Results

### Global cellular landscape in CRC

**Figure 1.**
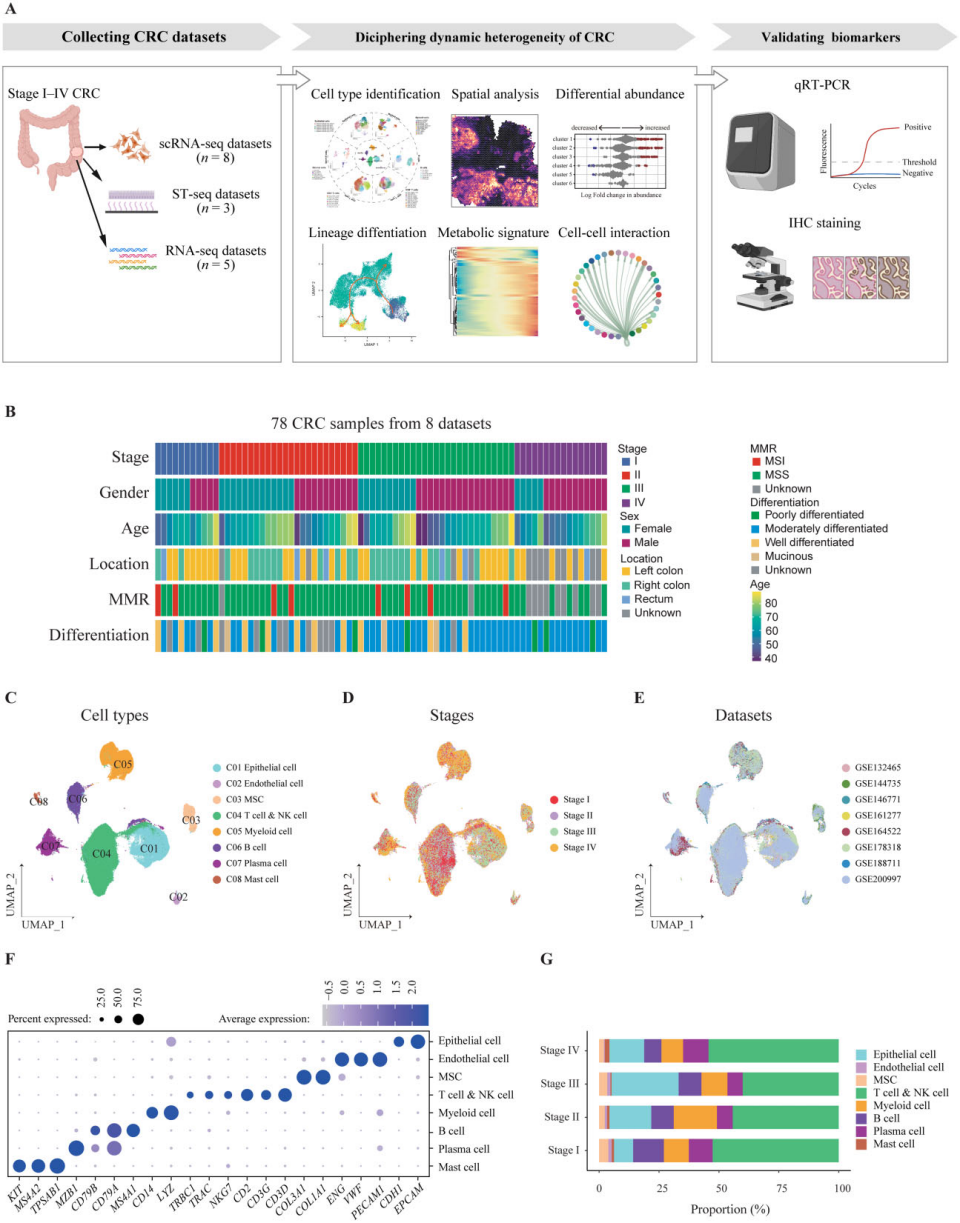
A single-cell transcriptomic atlas of tumor tissues from patients with stage I**–**IV CRC. (A) Graphic overview of this study design. A total of 16 data sets of scRNA-seq, ST-seq, and RNA-seq were collected to perform a comprehensive analysis to unveil dynamic heterogeneity during CRC progression. qRT–PCR and IHC staining were used to validate biomarkers associated with tumor progression. (B) Clinical characteristics of patients with CRC enrolled for scRNA-seq in this study. (C) Clusters, (D) stage information, and (E) original data sets of cells are shown in UMAP plots. (F) Dot plots illustrate the average expression of representative markers in indicated cell clusters. The dot size represents the percentage of cells expressing these markers and the dot color indicates the expression intensity. (G) Bar plot demonstrating the proportion of eight cell types in CRC tissues with indicated stage. CRC, colorectal cancer; scRNA-seq, single-cell RNA sequencing; ST-seq, spatial transcription sequencing; qRT–PCR, quantitative reverse transcription polymerase chain reaction; H & E, hematoxylin and eosin; IHC, immunohistochemistry; UMAP, Uniform Manifold Approximation and Projection.

The global cellular landscape in stage I–IV CRC was assessed using an analytic work flow, which consisted of the collection of sequencing data sets of CRC, deconvolution of tumor heterogeneity by bioinformatic analyses, followed by validation (Figure 1A). Eight data sets (GSE161277, GSE132465, GSE144735, GSE146771, GSE164522, GSE178318, GSE188711, GSE200997) with 78 tumor samples, including 11 stage I, 24 stage II, 27 stage III, and 16 stage IV CRC, were collected for subsequent analyses (Supplementary Table 2). Clinical information is provided in Figure 1B. After quality-control filtering and removal of any batch effect, 214,058 cells remained, including 38,811 epithelial cells, 167,667 immune cells, and 7,580 stromal cells, which were further subclustered (Figure 1C). Stage information and original data sets shown in a UMAP plot demonstrated successful removal of any batch effect (Figure 1D and E). The expression of representative markers of epithelial cells, endothelial cells, mesenchymal stromal cells (MSC), T cells and natural killer (NK) cells, myeloid cells, B cells, plasma cells, and mast cells are illustrated in Figure 1F. The proportions of each major cell type were different among patients with stage I–IV CRC (Figure 1G).

### The dynamics of malignant epithelial cells in CRC

**Figure 2.**
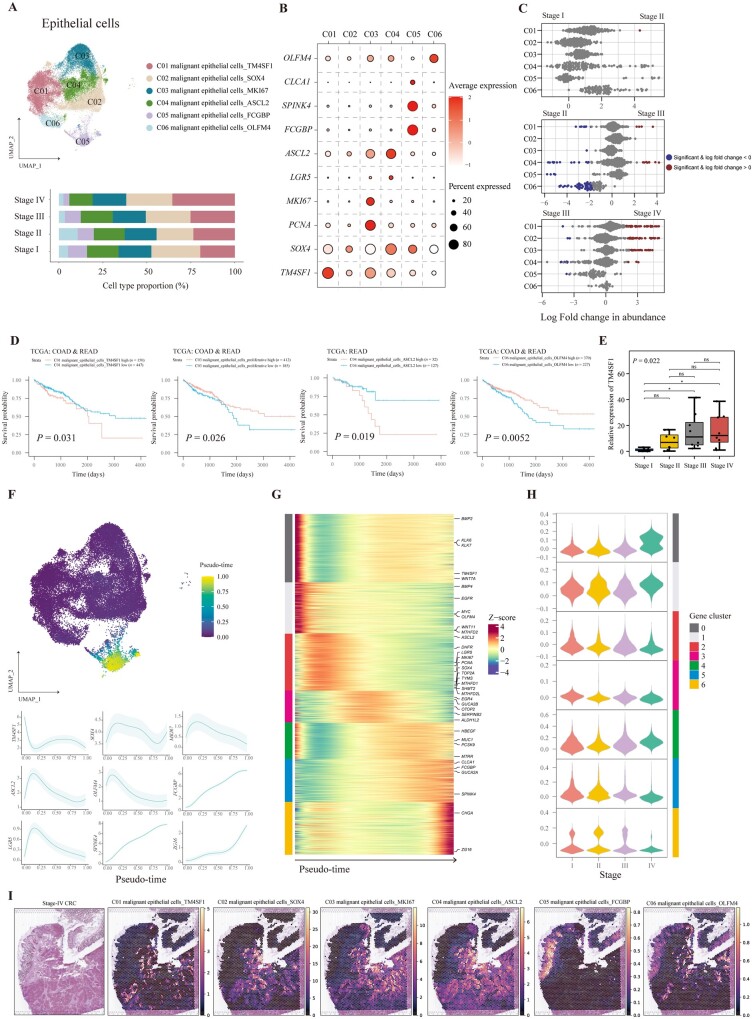
Characterization of malignant epithelial cells in CRC tissues with different stages. (A) UMAP plot and bar plot showing the composition of malignant epithelial cells colored by clusters. (B) Dot plots illustrating the average expression of representative markers in malignant epithelial cell types. The dot size represents the percentage of cells expressing these markers and the dot color indicates the expression intensity. (C) Beeswarm plots demonstrating the fold change of the cell abundance of each malignant epithelial cell type across different stages. Red and blue colors indicate significant differential abundance (Spatial FDR 10%). (D) Kaplan–Meier curves illustrating the OS for patients from TCGA–COAD and READ stratified by high and low infiltration of indicated malignant epithelial clusters. The *P*-value was calculated using the log-rank test. (E) Relative expression of TM4SF1 in CRC tissues validated by qRT–PCR with eight cases in each stage. Data are shown by median with interquartile range. *P*-value was calculated by using one-way ANOVA and Tukey's post hoc test; ns, *P *>* *0.05; *P <* *0.05. (F) Pseudo-time of each malignant epithelial cell imputed by Palantir is shown in the UMAP plot and the trends of the expression of representative markers are plotted. The data are shown as mean ± standard deviation. (G) Heat map showing the pseudo-time-smoothed expression of 2,000 highly variable genes of malignant epithelial cells. The color bars on the left side represent gene clusters, as in (H). (H) Violin plots showing expression of each gene cluster in malignant epithelial cells from CRC tissues with different stages. The color bars on the right side represent gene clusters. (I) Spatial abundance of six malignant cell types estimated using cell2location shown on a slice of stage IV CRC tissue with the corresponding image of H & E staining. CRC, colorectal cancer; UMAP, Uniform Manifold Approximation and Projection; FDR, false discovery rate; TCGA, The Cancer Genome Atlas; qRT–PCR, quantitative reverse transcription polymerase chain reaction; H & E, hematoxylin and eosin.

We further clustered 38,811 epithelial cells into six subtypes (C01–C06) (Figure 2A). To avoid contamination of normal epithelial cells when sampling tumor tissues, all epithelial cells were proven to be malignant by analysing the different chromosomal patterns of the copy-number variation compared with normal epithelial cells (Supplementary Figure 2A). Differential expression analysis found highly expressed markers that were mainly stem-cell-associated and epithelial-lineage-associated genes, specifically *TM4SF1* for C01; *SOX4* for C02; *MKI67* and *PCNA* for C03; *ASCL2* and *LGR5* for C04; *CLCA1*, *SPINK4*, and *FCGBP* for C05; and *OLFM4* for C06 (Figure 2B). Next, we analysed the differential abundance of six malignant epithelial cell types across different stages to unveil tumor-progression-associated cell types. Interestingly, we found that ASCL2^+^ malignant epithelial cells, FCGBP^+^ malignant epithelial cells, and OLFM4^+^ malignant epithelial cells were decreased in stage III samples compared with their stage II counterparts. Moreover, TM4SF1^+^ malignant epithelial cells, SOX4^+^ malignant epithelial cells, and MKI67^+^ malignant epithelial cells were significantly increased in stage IV samples in comparison with stage III samples (Figure 2C). These results indicated that the expression level of stem cells and epithelial-lineage-associated genes was dynamic during evolvement from stages I to IV, and stemness-associated genes may contribute to the progression and metastasis of CRC.

The evaluation of the impact of malignant epithelial cell composition on survival for patients with CRC could contribute to a better understanding of their biological behaviors. Therefore, we applied CIBERSORTx using scRNA-seq data to deconvolute the fraction of all cell types for RNA-seq data from TCGA or GEO, including TCGA**–**COAD, TCGA**–**READ, GSE17536, GSE17537, and GSE39582. We also calculated scores by GSVA using signatures of well-defined clusters to validate CIBERSORTx results. As a result, most of the fractions of cell types deconvoluted by using CIBERSORTx were positively correlated with GSVA scores, except for monocytes, NK cells, and CD4^+^ Treg, which were removed from survival analysis (Supplementary Figure 1). As for these malignant epithelial cells, we found that patients with a higher fraction of TM4SF1^+^ or ASCL2^+^ malignant epithelial cells had a lower OS rate, while patients with a higher percentage of MKI67^+^ or OLFM4^+^ malignant epithelial cells had a better prognosis (Figure 2D). These results were consistent with our results of abundance analysis, such as TM4SF1^+^ malignant epithelial cells, which were increased in patients with stage IV CRC and correlated with unfavorable prognoses. In addition, OLFM4^+^ malignant epithelial cells were decreased in patients with stage III CRC and exhibited high OS rates.

We further analysed the pathway enrichment of these six malignant epithelial cell types (Supplementary Figure 2B). GO analysis indicated enriched pathways including blood vessel remodeling function for TM4SF1^+^ malignant epithelial cells, regulation of apoptosis and fibroblast proliferation for SOX4^+^ malignant epithelial cells, DNA replication for proliferative malignant epithelial cells, regulation of the Wnt signaling pathway for ASCL2^+^ malignant epithelial cells, and ion metabolism for FCGBP^+^ malignant epithelial cells. The enriched pathways indicated that EMT occurred in malignant epithelial cells. We calculated the EMT score for each epithelial cell type, suggesting the ability of metastasis. As a result, TM4SF1^+^ malignant epithelial cells had the highest EMT score in these six clusters (Supplementary Figure 2C). We further used qRT–PCR to validate expression of *TM4SF1* in each stage of CRC tissue by using an independent cohort, which proved that *TM4SF1* was upregulated in advanced CRC (Figure 2E).

Next, we analysed the trajectory of malignant epithelial cells (Figure 2F). To validate the accuracy of the trajectory, CytoTRACE was applied, which also demonstrated the differentiation potential of cell types. Palantir indicated that FCGBP^+^ malignant epithelial cells were the most differentiated while CytoTRACE results also revealed those cells with the lowest scores (Supplementary Figure 2D and E). The trends of gene expression revealed that cells were ranged according to the pseudo-time from increased expression of *TM4SF1* with a high EMT score to stemness and followed by mature epithelial signatures (Figure 2G). We further defined seven gene clusters that were associated with trajectory and plotted the global expression level of these gene clusters in malignant epithelial cells for each stage (Figure 2G and H). Interestingly, except for gene cluster 4, malignant epithelial cells showed a high expression pattern of gene cluster 0 in stage IV CRC, and this pattern was gradually inverted for gene clusters 1, 2, 3, 5, and 6. Therefore, expression of gene clusters seemed to be gradually activated when CRC evolved from stages I to IV. GO analysis results indicated enriched pathways for these gene clusters (Supplementary Figure 2F). For example, metabolic pathways such as energy metabolism and O-glycan metabolism were activated in cells with a high expression of gene clusters 6 and 5, which might represent relatively normal epithelial function, while gene cluster 2 indicated proliferation of tumor cells and gene cluster 1 signified a pathway responding to decreased oxygen levels. Finally, gene cluster 0 indicated a pathway about the negative regulation of cell adhesion, which played an important role in metastasis. These enriched pathways of gene clusters elaborated key regulations in malignant epithelial cells during CRC progression and metastasis.

To have a deeper understanding of the biological behaviors of these six malignant epithelial clusters in CRC, we evaluated their abundance in a spatial context. We found that C01–C03 were more abundant in the slice from stage IV CRC, while C04–C06 were enriched in a stage II CRC sample, in line with the results of scRNA-seq (Figure 2I and Supplementary Figure 2G).

In general, a combination of scRNA-seq and ST-seq data showed the dynamic features of malignant epithelial cells in CRC and *TM4SF1* were highly expressed in malignant epithelial cells from advanced CRC, which could be used as a therapeutic target and prognostic indicator.

### The infiltration of CXCL12^+^ cancer-associated fibroblasts was increased in advanced CRC

**Figure 3.**
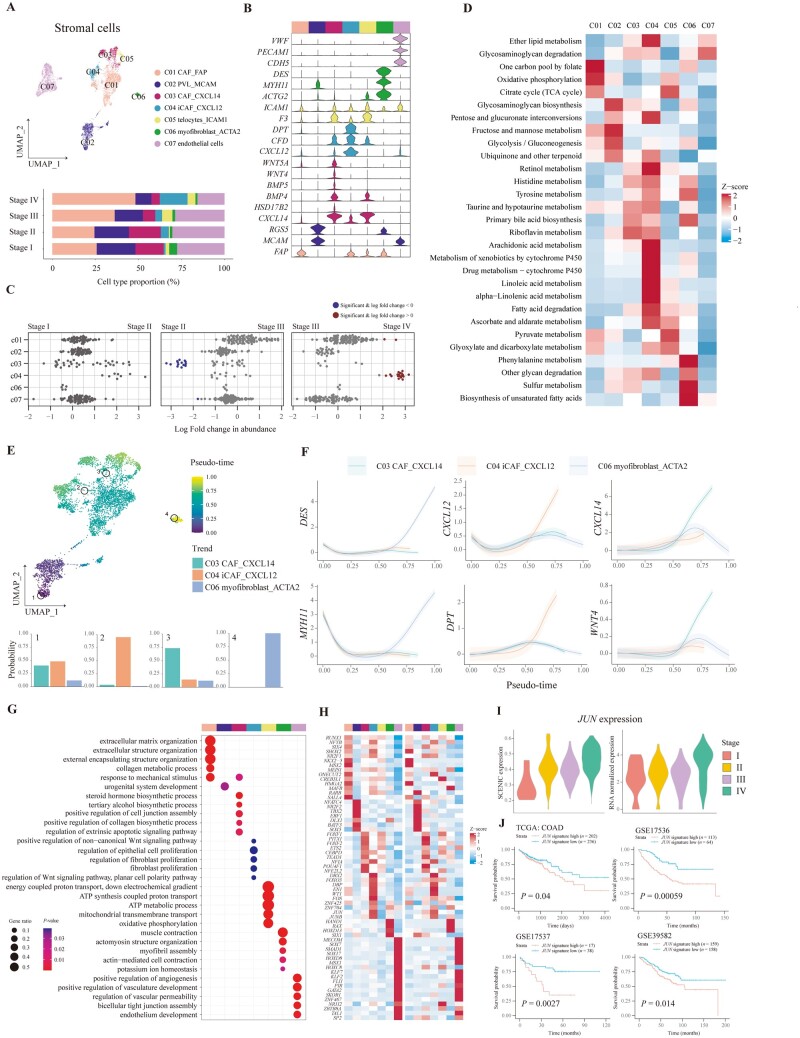
Characterization of stromal cells in different stages of CRC. (A) Composition of stromal cells shown in a UMAP plot and bar plot. (B) Violin plots showing the expression of representative markers of stromal cells. The color bars in the top represent stromal cell types, as in (A). (C) Beeswarm plots demonstrating the fold change of the cell abundance of each stromal cell type across different stages. Red and blue colors indicate significant differential abundance (Spatial FDR 10%). (D) Heat map illustrating the metabolism activity of stromal cell types. (E) Pseudo-time of each stromal cell shown in a UMAP plot with specific cells highlighted. Terminal state probability distributions of highlighted cells are visualized using bar plots. (F) The gene expression trends along stromal lineages are plotted. The data are shown as mean ± standard deviation. (G) Functional enrichment analysis of upregulated genes in each stromal cluster performed by using GO analysis. The color bars indicate stromal cell clusters, as in (A). (H) Heat maps illustrating the relative expression of top transcription factors predicted by using pySCENIC (left-side heat map) and in RNA level (right-side heat map). The color bars indicate stromal cell clusters, as in (A). (I) Violin plots showing the JUN-regulon expression of CXCL12^+^ iCAF in each stage of CRC predicted by using pySCENIC (left-side plot) and in RNA level (right-side plot). (J) Kaplan–Meier curves illustrating the OS for patients stratified by high and low expression of JUN-regulated genes using TCGA and GEO survival data. The *P*-value was calculated using the log-rank test. CRC, colorectal cancer; CAF, cancer-associated fibroblast; iCAF, inflammatory CAF; PVL, perivascular-like; UMAP, Uniform Manifold Approximation and Projection; FDR, false discovery rate; GO, gene ontology. TCGA, The Cancer Genome Atlas; GEO, Gene Expression Omnibus.

We classified 7,580 stromal cells into seven clusters, including endothelial cells expressing *PECAM1* and *CDH5*, as well as six other MSC types including FAP^+^ cancer-associated fibroblast, MCAM^+^ perivascular-like cells (PVL), CXCL14^+^ cancer-associated fibroblast (CAF), CXCL12^+^ inflammatory CAF (iCAF), ICAM1^+^ telocyte, and ACTG2^+^ myofibroblast (Figure 3A and B). To assess the dynamics of stromal cells along with CRC progression, differential abundance was analysed, indicating that CXCL14^+^ CAF was significantly decreased in stage III CRC tissues compared with stage II CRC tissues, and CXCL12^+^ iCAF was dramatically increased in stage IV CRC tissues compared with stage III CRC tissues (Figure 3C). We also detected the spatial distribution of all stromal cells in TME or the border of the tumor (Supplementary Figure 3A). FAP^+^ CAF and MCAM^+^ PVL were the most abundant CAF in CRC tissue, in agreement with results of scRNA-seq (Figure 3A). As for their effect on the survival of patients with CRC, we found that patients with a higher fraction of stromal cell types exhibited a lower OS rate (Supplementary Figure 3B–F).

Since CAF played a significant role in the metabolic reprogramming of tumor cells through interactions between CAF and tumor cells, we inquired into the metabolic signatures of stromal cells in the TME of CRC tissues (Figure 3D). As a result, FAP^+^ CAF was demonstrated with the highest activation of oxidative phosphorylation, MCAM^+^ PVL was indicated with the highest score of glycolysis and gluconeogenesis, and ICAM1^+^ telocyte showed the highest activity of the citrate cycle. Furthermore, drug metabolism was activated in CXCL12^+^ iCAF, which might be the potential mechanism of chemotherapy resistance in patients with CRC.

Next, we traced the lineage of MSC in CRC to infer their origin and division. Palantir demonstrated that MSC was differentiated from MCAM^+^ PVL to three other branches: CXCL14^+^ CAF, CXCL12^+^ iCAF, and ACTA2^+^ myofibroblast (Figure 3E), validated by the expression of lineage markers in the differential trajectory (Figure 3F). We also constructed the differentiation trajectory of MSC by using monocle3, which showed a similar differential trajectory (Supplementary Figure 3G and H). Functions of stromal cells were revealed by using GO analysis (Figure 3G). It was shown that FAP^+^ CAF expressed signatures of extracellular matrix organization and CXCL14^+^ CAF had several activated biosynthetic processes expressed including collagen biosynthesis, while CXCL12^+^ CAF was associated with the regulation of fibroblast proliferation. We also applied SCENIC to infer the transcription factor regulation of stromal cells. Associated regulons were identified for each stromal cluster, when these regulons could also distinctly cluster stromal cells, indicating that the activity of transcription factors could truly represent biological functions of MSC in regulon space (Figure 3H and Supplementary Figure 3I). It was found that transcription factor *JUN* was highly expressed in CXCL12^+^ iCAF, which has been reported to promote fibrosis. It was proven by upregulated activation of the regulon or mRNA expression of *JUN* of CXCL12^+^ iCAF in the tumor tissues of patients with advanced-stage CRC (Figure 3I). We further calculated the expression of genes regulated by transcription factor *JUN* in RNA-seq data sets and found that patients with a higher *JUN* signature showed worse prognosis (Figure 3J). Therefore, CXCL12^+^ iCAF was increased in the stage IV CRC and demonstrated with higher expression of *JUN*, which could contribute to fibrosis.

### The infiltration of CD4^+^ Treg was increased in stage IV CRC with a high metabolic signature

**Figure 4.**
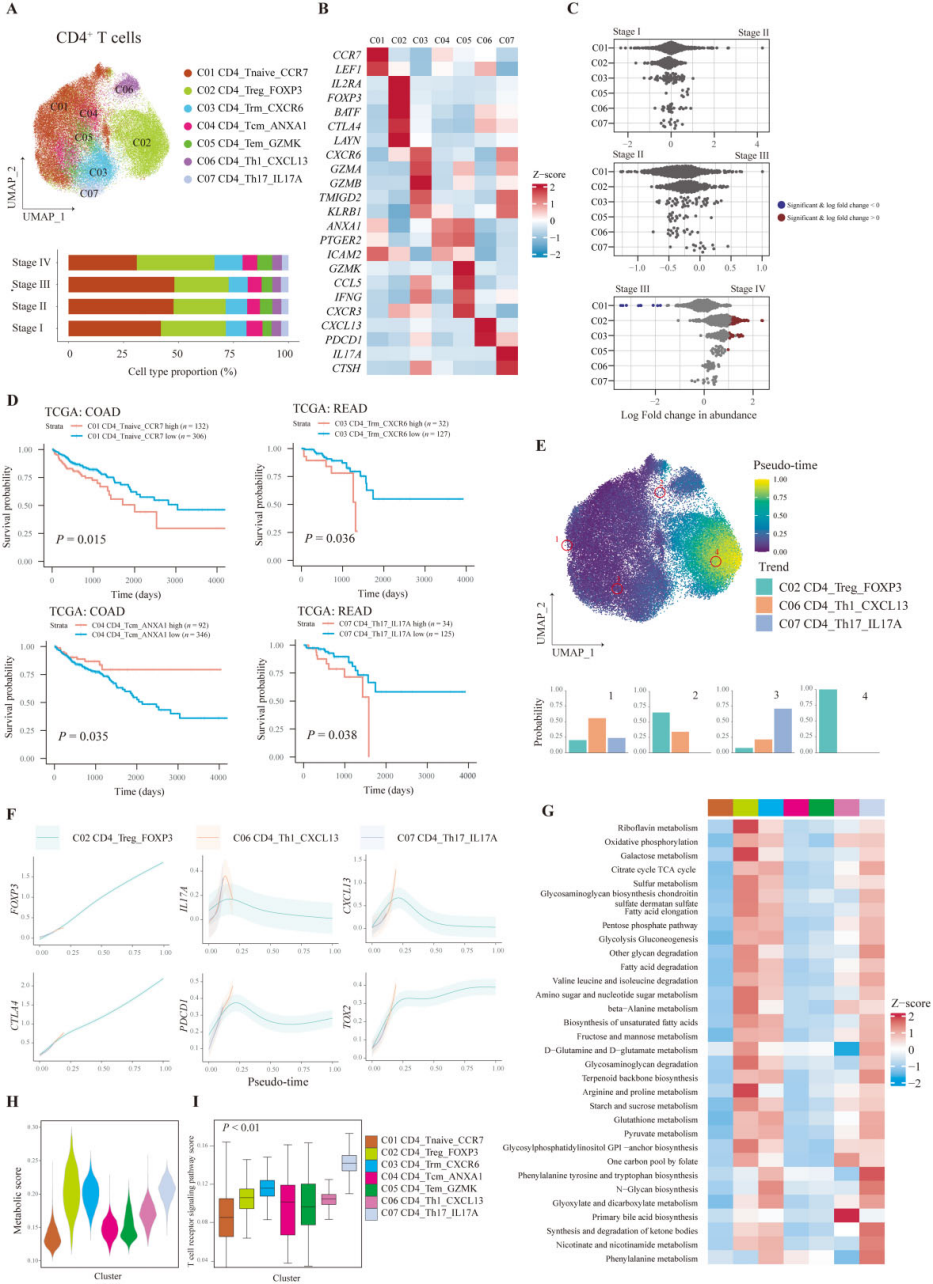
Characterization of CD4^+^ T cells in CRC tissues with different stages. (A) UMAP plot showing the seven main CD4^+^ T-cell subtypes. Bar plot indicates the proportion of CD4^+^ T cells in different stages of CRC tissues. (B) Relative expression of representative markers of CD4^+^ T-cell subtypes. (C) Beeswarm plots of fold change of cell abundance for each CD4^+^ T cluster across different stages. Red and blue colors indicate significant differential abundance (Spatial FDR 10%). (D) Kaplan–Meier curves of patients from TCGA–COAD or READ stratified by high and low infiltration of CD4^+^ T-cell types. A two-sided log-rank test was used to assess statistical significance. (E) Pseudo-time of CD4^+^ T cells shown in a UMAP plot with specific cells highlighted. Terminal state probability distributions of highlighted cells are visualized using bar plots. (F) The gene expression trends of representative markers along CD4^+^ T-cell lineages are plotted. The data are shown as mean ± standard deviation. (G) The metabolism activity of CD4^+^ T-cell clusters are shown in a heat map. The colors of the top bars indicate CD4^+^ T clusters, as in (A). Violin plots demonstrating the (H) metabolism activity and (I) TCR signaling of each CD4^+^ T-cell type. One-way ANOVA was performed to assess statistical significance. CRC, colorectal cancer; Trm, memory T cell; Tcm, central memory T cell; Tem, effector memory T cell; UMAP, Uniform Manifold Approximation and Projection; FDR, false discovery rate; TCGA, The Cancer Genome Atlas; COAD, colon adenocarcinoma; READ, rectum adenocarcinoma; TCR, T-cell receptor.

We obtained 51,600 CD4^+^ T cells and 7 subtypes were finally identified, including CCR7^+^ naive T cells, FOXP3^+^ Treg, CXCR6^+^ resident memory T cells (Trm), ANXA1^+^ central memory T cells (Tcm), GZMK^+^ effector memory T cells (Tem), CXCL13^+^ Th1 cells, and IL17A^+^ Th17 cells (Figure 4A). The representative markers for the indicated CD4^+^ T-cell cluster are shown at Figure 4B. To reveal alteration of CD4^+^ T cells during CRC progression, the differential abundance across stage I–IV CRC was analysed. The infiltration of CD4^+^ naive T cells was decreased in stage IV CRC tissues, while the proportion of Treg and Trm cells was increased in stage IV CRC tissues compared with those in stage III CRC tissues (Figure 4C). As for cell abundance in slices, ST-seq showed that the abundance of all CD4^+^ T-cell subsets was low and infiltrated in the surrounding area of tumors (Supplementary Figure 4A). However, as for prognosis, only a higher fraction of CD4^+^ naive T cells, CD4^+^ Trm cells, and Th17 cells as well as a lower fraction of CD4^+^ Tcm cells was associated with a lower OS rate (Figure 4D).

To better understand the function and differentiation state of CD4^+^ T cells in CRC, we analysed the transcriptomic trajectory of CD4^+^ T cells. Three major branches comprising CD4^+^ Treg, Th1, and Th17 were recognized (Figure 4E). Lineage-associated genes for these branches are shown at Figure 4F. We further validated the results of Palantir by using monocle3, which also identified the same three branches (Supplementary Figure 4B and C). Next, we observed the heterogeneous expression of immune checkpoints in the branching trajectory of CD4^+^ T cells. *PDCD1* and *TOX2* were increasingly expressed in CXCL13^+^ Th1 cells, while *CTLA4* was highly expressed in CD4^+^ Treg cells (Figure 4F). These results indicated that different terminal states of CD4^+^ T cells could have different expression levels of immune checkpoints and therefore immune checkpoints blockade could selectively affect different branches of CD4^+^ T cells.

T-cell activation and exhaustion play an important role in antitumor immunity. Since we have unveiled that exhaustion-associated markers such as *CTLA4*, *PDCD1*, and *TOX2* were upregulated in the terminal states of CD4^+^ T cells, we further inquired into the activated states of CD4^+^ T cells, which could be directly reflected by the metabolism and activation of the TCR signaling pathway. Metabolic analysis indicated that numerous metabolic pathways were upregulated in CD4^+^ Treg, Trm, Th1 cells, and Th17 cells (Figure 4G and H). CD4^+^ T-cell clusters that presented with a high metabolic score were found to have a high TCR signaling pathway score (Figure 4I). Therefore, during differentiation, TCR signaling, exhaustion-associated markers, and metabolic pathways of CD4^+^ T cells were upregulated.

### The majority of CD8^+^ T cells in CRC TME were differentiated into proliferative and exhausted terminal states

**Figure 5.**
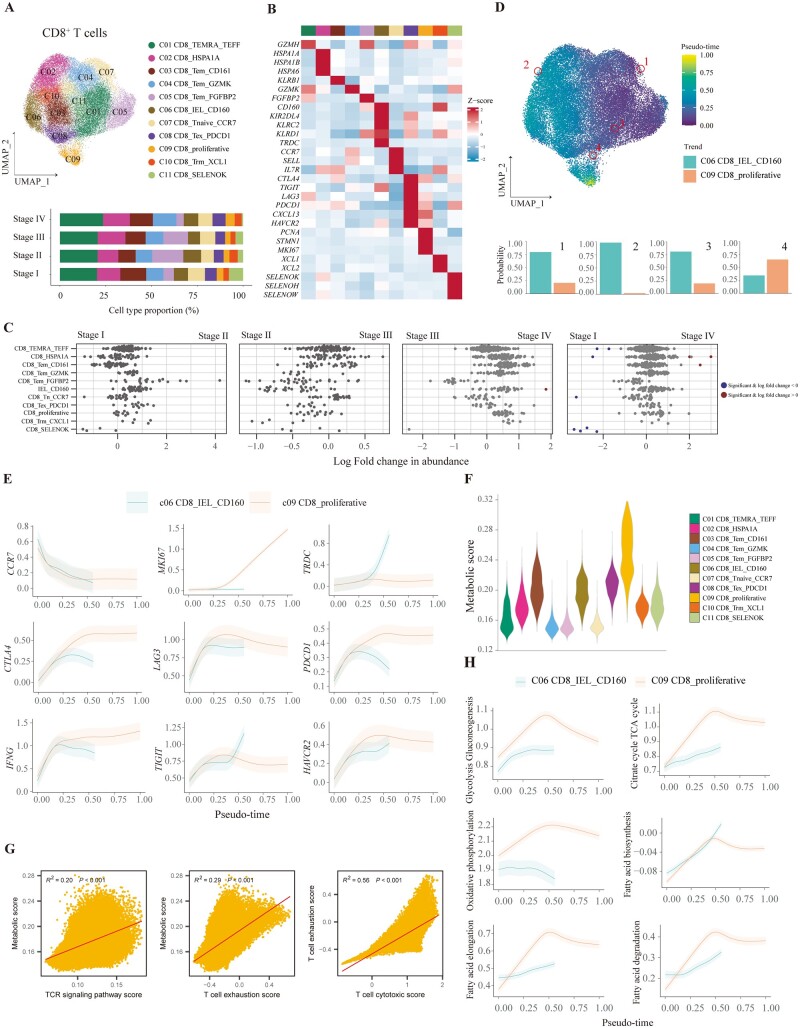
CD8^+^ T cells differentiate into terminal state with high exhaustive and metabolic signatures in CRC tissues. (A) UMAP plot and bar plot showing the composition of CD8^+^ T cells. (B) Heat map plot depicting the relative expression levels of representative markers for CD8^+^ T-cell types. The top color bars indicate CD8^+^ T-cell clusters, as in (A). (C) Beeswarm plots of fold change in cell abundance for each CD8^+^ T cell across different stages. Red and blue colors indicate significant differential abundance (Spatial FDR 10%). (D) UMAP plot illustrating pseudo-time of CD8^+^ T cells with specific cells highlighted. The probability of differentiation to two terminal states for highlighted CD8^+^ T cells is shown in bar plots. (E) The trends of the expression of selected markers for all CD8^+^ T lineages are plotted. The data are shown as mean ± standard deviation. (F) Violin plot depicting the global metabolic activity of each CD8^+^ T-cell type. (G) Scatter plots demonstrating the correlation between metabolism activity and TCR signaling (left), correlation between metabolism activity and T-cell exhaustion score (middle), and correlation between T-cell cytotoxic score and T-cell exhaustion score (right) for all CD8^+^ T cells. The error band indicates the 95% confidence interval. (H) The trends of metabolism activity are shown for each differentiation direction of CD8^+^ T cells. The data are shown as mean ± standard deviation. CRC, colorectal cancer; Trm, memory T cell; Tem, effector memory T cell; TEMRA/TEFF, recently activated effector memory or effector T cell; IEL, intraepithelial lymphocyte; Tex, exhausted T cell; UMAP, Uniform Manifold Approximation and Projection; FDR, false discovery rate; TCR, T-cell receptor; TCA, tricarboxylic acid.

To elucidate the activation and exhaustion signatures of CD8^+^ T cells during CRC progression, we identified 11 clusters from a total of 38,180 CD8^+^ T cells, comprising GZMH^+^ recently activated effector memory or effector T cells (TEMRA/TEFF), HSPA1A^+^ T cells, CD161^+^ Tem, GZMK^+^ Tem, FGFBP2^+^ Tem, CD160^+^ intraepithelial lymphocytes (IEL), CCR7^+^ naive T cells, PDCD1^+^ exhausted T cells (Tex), MKI67^+^ proliferative T cells, XCL1^+^ Trm, and SELENOK^+^ T cells (Figure 5A and B). Next, differential abundance analysis indicated that only CD8^+^ SELENOK^+^ T cells were increased in stage IV CRC compared with stage I CRC, suggesting insignificant alteration of the proportion of CD8^+^ T cells in TME during CRC progression (Figure 5C). The abundance of CD8^+^ T cells was shown to be low in spatial transcription (Supplementary Figure 5A). We further identified the biological function of CD8^+^ T cells. GO analysis demonstrated that the functions of CD8^+^ SELENOK^+^ T cells corresponded to energy metabolism, such as ATP synthesis and mitochondrial transmembrane transport (Supplementary Figure 5B). The functions of other CD8^+^ T-cell subtypes were mainly related to activation and cytotoxicity. These results hinted that energy metabolism was activated in CD8^+^ SELENOK^+^ T cells.

We next constructed the differentiation trajectory of CD8^+^ T cells. There were two branches recognized by the Palantir algorithm: CD8^+^ CD160^+^ IEL and CD8^+^ proliferative T cells (Figure 5D). Furthermore, terminal proliferative CD8^+^ T cells highly expressed inhibitory receptors, such as *CTLA4*, *LAG3*, *PDCD1*, *TIGIT*, *HAVCR2*, and cytotoxic marker *IFNG* (Figure 5E). These immune checkpoints were also heterogeneously expressed in these two branches of CD8^+^ T cells: *TIGIT* was highly expressed in CD160^+^ IEL, while the expression of other inhibitory receptors such as *LAG3*, *PDCD1*, and *HAVCR2* was dominant in CD8^+^ proliferative T cells.

Since tumor-infiltrating CD8^+^ T cells became more exhausted during differentiation in CRC, we inquired into their metabolic states, cytotoxic function, and exhaustive signature. CD8^+^ proliferative T cells, CD161^+^ Tem cells, CD160^+^ IEL, and PDCD1^+^ Tex cells were demonstrated to have more activated metabolic states compared with other T cells, while oxidative phosphorylation was upregulated in SELENOK^+^ CD8^+^ T cells, which was consistent with GO analysis (Figure 5F and Supplementary Figure 5C). We also inquired into the relationship between the activation of the TCR pathway and the metabolic profile. A positive relationship between TCR signaling and metabolic state was observed in CD8^+^ T cells (Figure 5G). Furthermore, T-cell exhaustion scores were also positively correlated with metabolic scores and T-cell cytotoxic scores. These results showed that the intensity of the dysfunctional signature of CD8^+^ T cells was associated with antitumor reactivity. Terminal exhausted CD8^+^ T cells in CRC were highly proliferating and dynamically differentiating. To have a deeper understanding of T-cell metabolism during differentiation, we analysed the metabolic states in the branching trajectory of CD8^+^ T cells (Supplementary Figure 5D and E). As for the branch of CD8^+^ CD160^+^ IEL, numerous metabolic pathways were upregulated during differentiation, except for oxidative phosphorylation (Figure 5H and Supplementary Figure 5D). However, during the process of differentiation to proliferative CD8^+^ T cells, metabolic states were increased and slightly downregulated at the terminal state in numerous pathways (Figure 5H and Supplementary Figure 5E). In particular, proliferative CD8^+^ T cells were more activated than CD160^+^ IEL in glycolysis, gluconeogenesis, Tricarboxylic acid (TCA) cycle, oxidative phosphorylation, and fatty acid metabolism (Figure 5H). Taken together, CD8^+^ T cells in CRC were differentiated to exhausted states together with proliferative and high metabolic signatures.

### The infiltration of IgA^+^ plasma cells and AICDA^+^ germinal center B cells were increased in stage IV CRC

**Figure 6.**
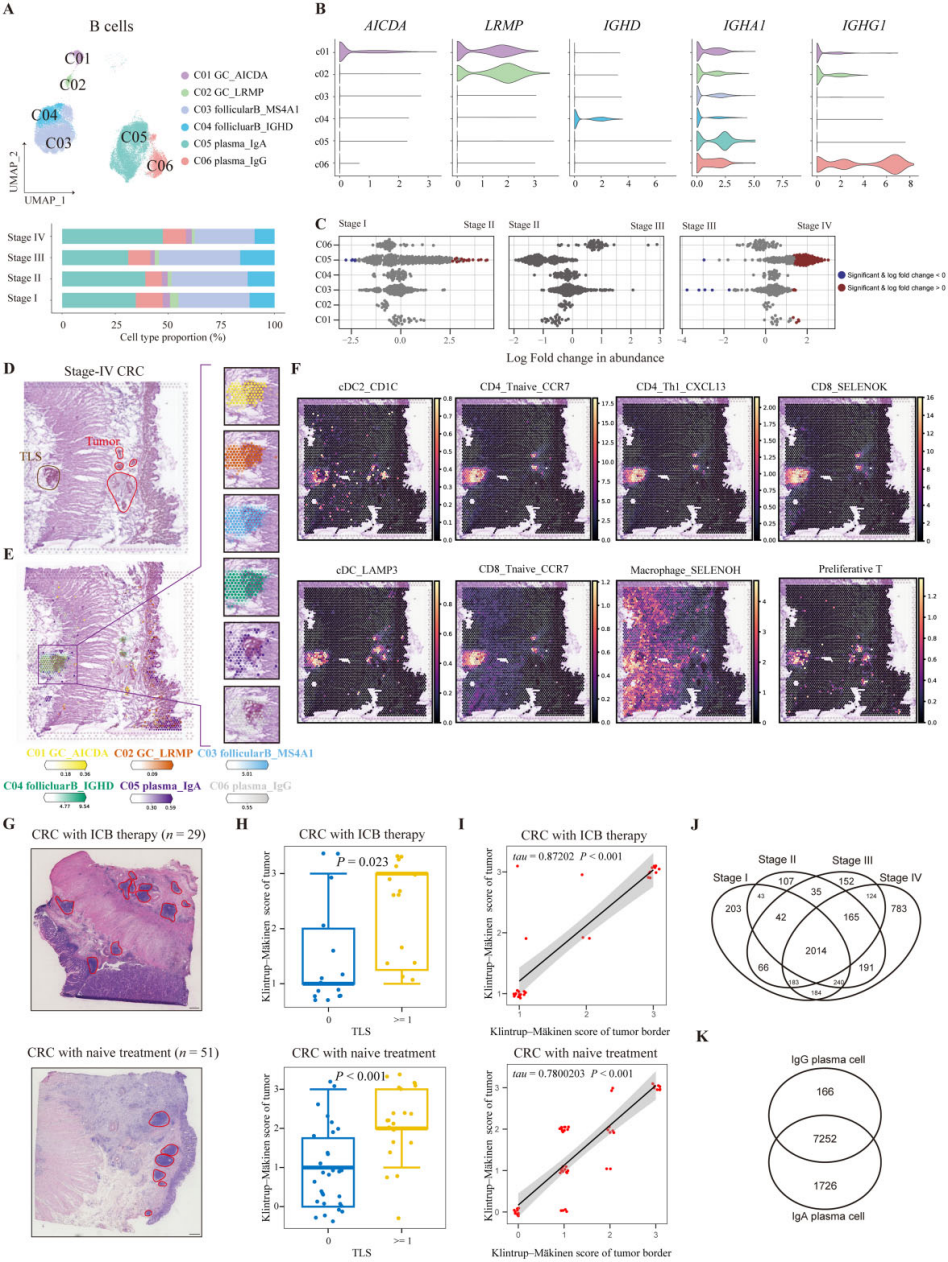
B cells were enriched in TLS and IgA–IgG antibody class switching occurred in CRC tissues. (A) UMAP plot and bar plot showing the composition of B cells. (B) Violin plot depicting expression of representative markers of B cells. (C) Beeswarm plots of fold change in cell abundance for B-cell types across different stages. Annotation by red and blue colors indicate significant differential abundance (Spatial FDR 10%). (D) The image of H & E staining from a slice of stage IV CRC tissue. Regions of TLS and tumor are annotated. (E) Spatial abundance of six B-cell types estimated by using cell2location is shown on a slice of stage IV CRC tissue with color gradient and interpolation. The region of TLS is annotated by a rectangle and the spatial abundance of B-cell types is shown on the right-side plots. (F) Spatial abundance revealing the enrichment of cell subtypes of T cells, DC, and macrophages in TLS. (G) Representative images of H & E staining in invasive margins of CRC tissues with or without anti-PD1 therapy. The regions annotated by red color indicate TLS. (H) Bar plots showing the distribution of Klintrup–Mäkinen scores of tumor regions according to the existence of TLS in patients with or without anti-PD1 therapy. Wilcoxon test was performed. (I) Correlation of Klintrup–Mäkinen scores between tumor border and tumor core of patients with or without PD-1 inhibitor. Kendall’tau and *P*-value were calculated. The error band indicates the 95% confidence interval. (J) Venn diagram illustrating the relationship of BCR between B cells from CRC tissues assigned with four stages. (K) Venn diagram showing the overlapped identified BCR between IgA^+^ plasma cells and IgG^+^ plasma cells. TLS, tertiary lymphoid structure; CRC, colorectal cancer; UMAP, Uniform Manifold Approximation and Projection; FDR, false discovery rate; H & E, hematoxylin and eosin; cDC, classical dendritic cell; BCR, B-cell receptor.

Here we obtained transcriptomes of 36,899 B cells from 78 patients with CRC followed by subclustering into six clusters, including two germinal center (GC) B-cell types, two follicular B-cell types, and two plasma cell types (Figure 6A and B). Differential abundance analysis showed that the proportion of AICDA^+^ GC B cells and IgA^+^ plasma cells was increased in stage IV CRC (Figure 6C). We analysed the B-cell abundance in the slices of tumors with/without tertiary lymphoid structures (TLS), including atopic lymphoid nodes or solitary lymphatic follicles (Figure 6D and E, and Supplementary Figure 6A). Considerable numbers of B cells and plasma cells were infiltrated in TME while they were more likely to be enriched in lymphoid organs, but only GC B cells were enriched in atopic lymph nodes. TLS located at TME were composed of aggregated T cells, mature DC, and B-cell follicles with GC and surrounded by plasma cells, which was evidenced by spatial transcription (Figure 6D–F). DC subsets including cDC2, LAMP3^+^ DC, T-cell subsets comprising naive CD4^+^ or CD8^+^ T cells, CXCL13^+^ Th1, CD8^+^ SELENOK^+^ T cells, proliferating T cells, and SELENOH^+^ macrophage were enriched in TLS. In particular, most of these cell types could be infiltrated into tumor regions, indicating the potential relation of immune cells between TLS and tumor (Figure 6E and F). To validate the relationship between TLS and the immune infiltration of TME, we utilized an independent cohort comprising 29 patients with anti-PD-1 therapy and surgery, and 51 patients with surgery to assess the Klintrup–Mäkinen score by using the H & E staining of CRC tissues or invasive margins (Figure 6G), when dichotomy was also applied to patients by the existence of TLS. As a result, we found that the existence of TLS was not relative to the stage or treatment with PD-1 inhibitors, but TLS could result in a higher immune infiltration in both tumors and invasive margins, which proved that TLS in TME could fuel immunity regardless of PD-1 inhibitors (Figure 6H and Supplementary Table 4). We also observed that the Klintrup–Mäkinen scores were positively correlated between tumors and invasive margins, suggesting that the global landscape of the immune reaction was connective (Figure 6I). Furthermore, Klintrup–Mäkinen scores were likely to be decreased as the tumor progressed from stage I to stage IV, suggesting dysfunction of immune regulation during CRC progression (Supplementary Table 5). Survival analysis of TCGA RNA-seq data indicated that a high expression of signatures of CD20^+^ B cells, IgA^+^ plasma cells, and IgG^+^ plasma cells was associated with a higher OS rate, suggesting the antitumor function of the B-cell lineage (Supplementary Figure 6B–D).

We further analysed the function of B-cell subtypes. It was shown that AICDA^+^ GC B cells were associated with cell proliferation while other B-cell subtypes expressed genes related to antigen processing and presentation (Supplementary Figure 6E). Metabolic pathway analysis revealed that follicular B cells were less active than GC and plasma cells, and metabolic states were dramatically different between GC and plasma cells (Supplementary Figure 6F and G). We also analysed the BCR signaling pathway and its relationship with metabolic states of B cells (Supplementary Figure 6H). Notably, the BCR signaling pathway was highly activated in GC and follicular B cells while it was decreased in plasma cells. LRMP^+^ GC B cells expressed higher BCR signaling than AICDA^+^ GC B cells when these two types of GC B cells showed different distributions, suggesting that AICDA^+^ and LRMP^+^ GC B cells play different roles in TLS and mucosa (Figure 6E and Supplementary Figure 6H). Moreover, metabolic scores of B cells were negatively correlated with activity of the BCR signaling pathway (Supplementary Figure 6I).

Since antibody class switching has been observed in several tumors, we further analysed the distribution of BCR and IgA–IgG switching in CRC. Since only variable regions could be sequenced in the 10x Genomics platform, we detected the paired variable region of light chain and heavy chain. As a result, most of paired variable regions of BCR could be detected in all stages of CRC, while stage IV CRC was composed of most BCR (Figure 6J). As shown by analysis based on variable regions of light chain, most BCR were shared across the tumor stages, and MS4A1^+^ follicular B cells and IgA^+^ plasma cells obtained the most diverse variable regions (Supplementary Figure 6J). In agreement with the results of different abundance, more BCR were detected in IgA^+^ plasma cells from stage IV CRC. As for paired variable regions shared by IgA and IgG, most BCR of IgG could be identified in IgA, suggesting the existence of antibody class switching from IgA to IgG (Figure 6K). IgG^+^ plasma cells were also more abundant than IgA^+^ plasma cells in tumor regions revealed by ST-seq (Supplementary Figure 6A). Altogether, our results unveiled that pairs of variable regions were enriched in stage IV CRC and that antibody class switching between IgA and IgG plasma cells was prevalent in CRC tissues.

### The infiltration of myeloid subsets was increased in stage IV CRC and colocalized with CD8^+^ T cells and tumor cells

**Figure 7.**
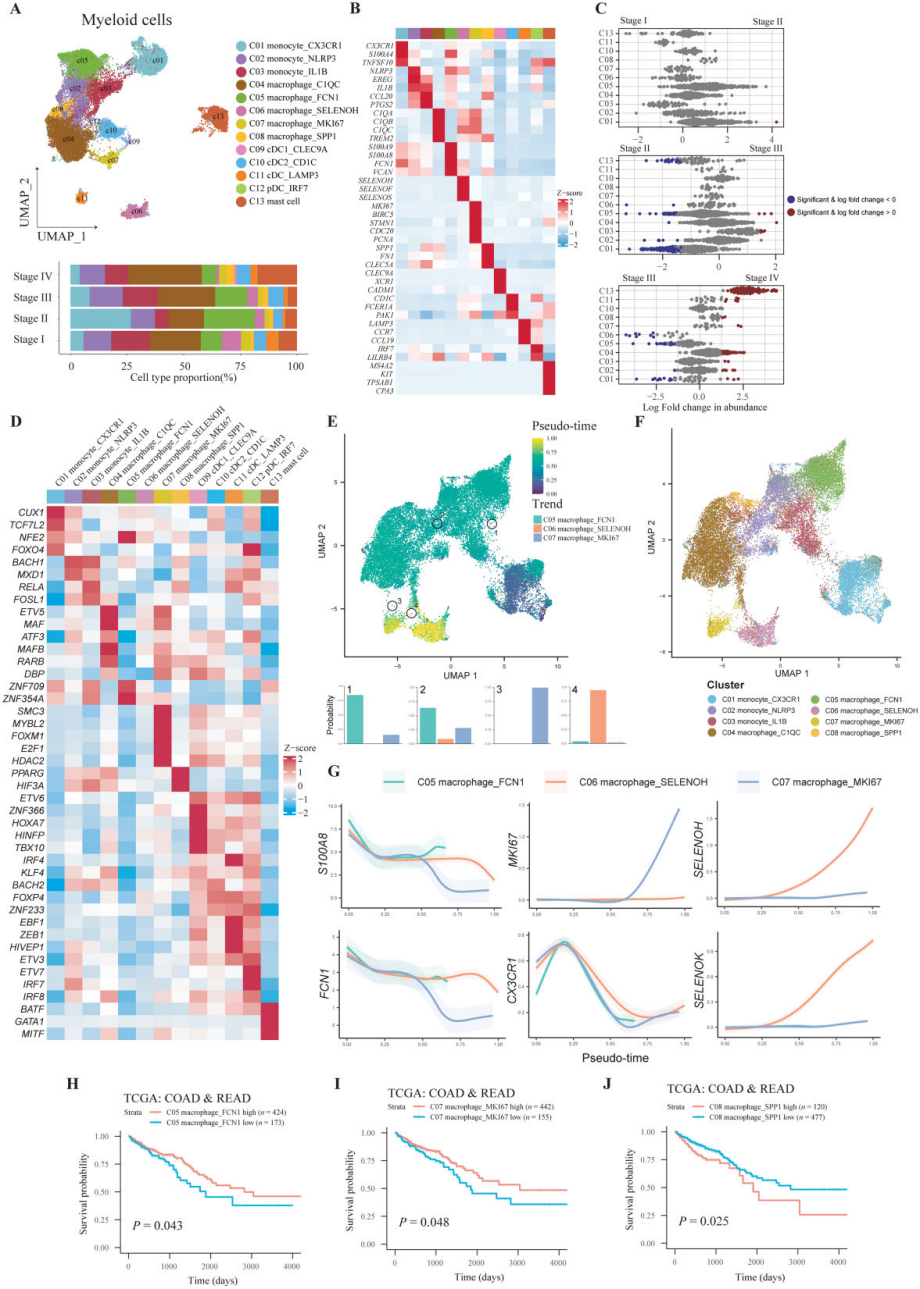
Characterization of myeloid cells in CRC tissues with different stages. (A) UMAP plot and bar plot showing the composition of myeloid cells. (B) Relative expression of representative markers of myeloid cells. (C) Beeswarm plots of fold change in cell abundance for myeloid cell types across different stages. Red and blue colors indicate significant differential abundance (Spatial FDR 10%). (D) Heat map demonstrating the relative expression of transcription factors predicted by using pySCENIC. (E) UMAP plot showing the pseudo-time of monocytes and macrophages with specific cells highlighted. Terminal state probability distributions of highlighted cells is revealed by bar plots. (F) Clusters of monocytes and macrophages are shown in a UMAP plot. (G) The expression trends of representative markers for all lineages of monocytes and macrophages. The data are shown as mean ± standard deviation. (H)–(J) Overall survival analysis for patients from TCGA–COAD and TCGA–READ stratified by low and high infiltration of (H) C05, (I) C07, and (J) C08 myeloid cells using Kaplan–Meier curves by two-sided log-rank test. CRC, colorectal cancer; cDC, classical dendritic cell; pDC, plasmacytoid dendritic cell; UMAP, Uniform Manifold Approximation and Projection; FDR, false discovery rate; TCGA, The Cancer Genome Atlas; COAD, colon adenocarcinoma; READ, rectum adenocarcinoma.

To reveal the alteration of components, antitumor immunity-associated transcription factors, and their spatial context of myeloid cells during CRC progression, we obtained 29,585 myeloid cells and finally 13 clusters were identified, including 3 monocyte subtypes (CX3CR1^+^ monocyte, NLRP3^+^ monocyte, and IL1B^+^ monocyte), 5 macrophage subtypes (C1QC^+^ macrophage, FCN1^+^ macrophage, SELENOH^+^ macrophage, MKI67^+^ macrophage, and SPP1^+^ macrophage), 4 DC subtypes (CLEC9A^+^ classical dendritic cell type 1 (cDC1), CD1C^+^ cDC2, LAMP3^+^ DC, and IRF7^+^ plasmacytoid dendritic cells (pDC)), and mast cell (KIT^+^ mast cell) (Figure 7A and B). Differential abundance analysis indicated that CX3CR1^+^ monocytes and FCN1^+^ macrophages were decreased from stage II to stage IV CRC, while C1QC^+^ macrophages and mast cells were increased in stage IV CRC as compared with stage III CRC (Figure 7C). SELENOH^+^ macrophage in stage IV CRC was less than that in stage III CRC. These results indicated that innate immunity was highly activated in stage IV CRC. Integrated analysis of scRNA-seq and ST-seq demonstrated that most myeloid cells were sparse in TME, while their location was also overlaid with T cells (Supplementary Figures 4A, 5A, and 7A).

Diverse functions highlight the heterogeneous and plastic nature of myeloid cells. We further analysed the function of each myeloid subset by using GO enrichment (Supplementary Figure 7B). CX3CR1^+^ monocytes were enriched with the pathway of leukocyte migration signaling, indicating chemotaxis of blood monocytes to TME. Expression of *NLRP3* and *IL1B* in monocytes indicated the response to lipopolysaccharides, therefore boosting subsequent activation of the NLRP3 inflammasome. Gene signatures of C1QC^+^ macrophages were related to complement activation and FCN1^+^ macrophages exhibited defense to fungus. The procedure of energy metabolism was activated in SELENOH^+^ macrophages, similarly to CD8^+^ SELENOK^+^ T cells. As for DC, pathways of antigen processing, the presentation of exogenous antigen, and the regulation of NK cells and T cells were enriched. We also applied SCENIC to inquire into the functional transcription factors for myeloid cells (Figure 7D). As a result, *FOXO4*, a transcription factor promoting an early inflammatory response, was upregulated in CX3CR1^+^ monocytes. *BACH1* and *MXD1* were highly expressed in NLRP3^+^ and IL1B^+^ monocytes. On the other hand, *MAF*, the key regulator of acute inflammatory responses, was identified in C1QC^+^ macrophage. As for MKI67^+^ macrophage, proliferation-associated transcription factor *SMC3* and *MYBL2* were highly expressed. *PPARG*, which was associated with angiogenesis, was identified in SPP1^+^ macrophage. Furthermore, *HIF3A*, a transcriptional regulator in adaptive response to low-oxygen tension, was also expressed in SPP1^+^ macrophage. As for transcription factors related to DC, most of them contributed to the development and maturation of DC, such as *ETV6* for cDC1, *IRF4* and *KLF4* for cDC2, and *IRF7* for pDC. On the other hand, *BATF*, *GATA1*, and *MIF*, which were important for mast-cell development, were also detected. In conclusion, myeloid cells were heterogeneous and pathway enrichment as well as transcriptional regulation analysis indicated their plasticity in TME.

Next, we analysed the metabolism of each subtype of myeloid cells in CRC (Supplementary Figure 7C and D). As a result, while monocle subtypes were less active in metabolic pathways, nearly all macrophages harbored higher metabolic activity except for FCN1^+^ macrophages. Moreover, C1QC^+^ macrophages as well as MKI67^+^ macrophages were highly activated in numerous metabolic pathways and these cells were increased in stage IV CRC, indicating that their metabolic reprogramming might contribute to metastasis.

We further inferred a differentiation trajectory of monocytes and macrophages in CRC. Both Palantir and the monocle3 algorithm identified three branches including FCN1^+^ macrophages, SELENOH^+^ macrophages, and MKI67^+^ macrophages (Figure 7E and F, and Supplementary Figure 7E and F). It was shown that the monocyte marker *CX3CR1* was downregulated during differentiation while branch-associated markers *S100A8*, *FCN1*, *MKI67*, *SELENOH*, and *SELENOK* were upregulated or maintained (Figure 7G). Integration with TCGA RNA-seq indicated that a high fraction of FCN1^+^ macrophages and MKI67^+^ macrophages in CRC were associated with a higher OS rate (Figure 7H and I). On the other hand, a high fraction of SPP1^+^ macrophages in CRC signified a lower OS rate (Figure 7J).

Myeloid cells could interact with tumor cells, stromal cells, and other immune cells, which exerted immunoregulatory functions. We generated a colocalization profile of different cell types in CRC. Regions annotated by colocalization profiles were similar to regions clustered by transcription and corresponded to H & E staining images likewise (Supplementary Figure 8A and B). We found that several myeloid cell types were colocalized with CD8^+^ T cells and DC, surrounding tumor cells in a stage IV CRC (Supplementary Figure 8A and C). Latent factor 3 was contributed by subsets of macrophages, DC, as well as T cells, and the distribution of latent factor 3 was near to latent factors 1, 5, and 6, which were enriched with tumor cells. On another slice from a stage III CRC, DC were colocalized with T cells as indicated by latent factor 6, which surrounded latent factor 8 enriched with proliferative malignant cells (Supplementary Figure 8B and D). These results indicated that the interaction between macrophages, DC, T cells, and tumor cells played an important role in antitumor immunity.

**Figure 8.**
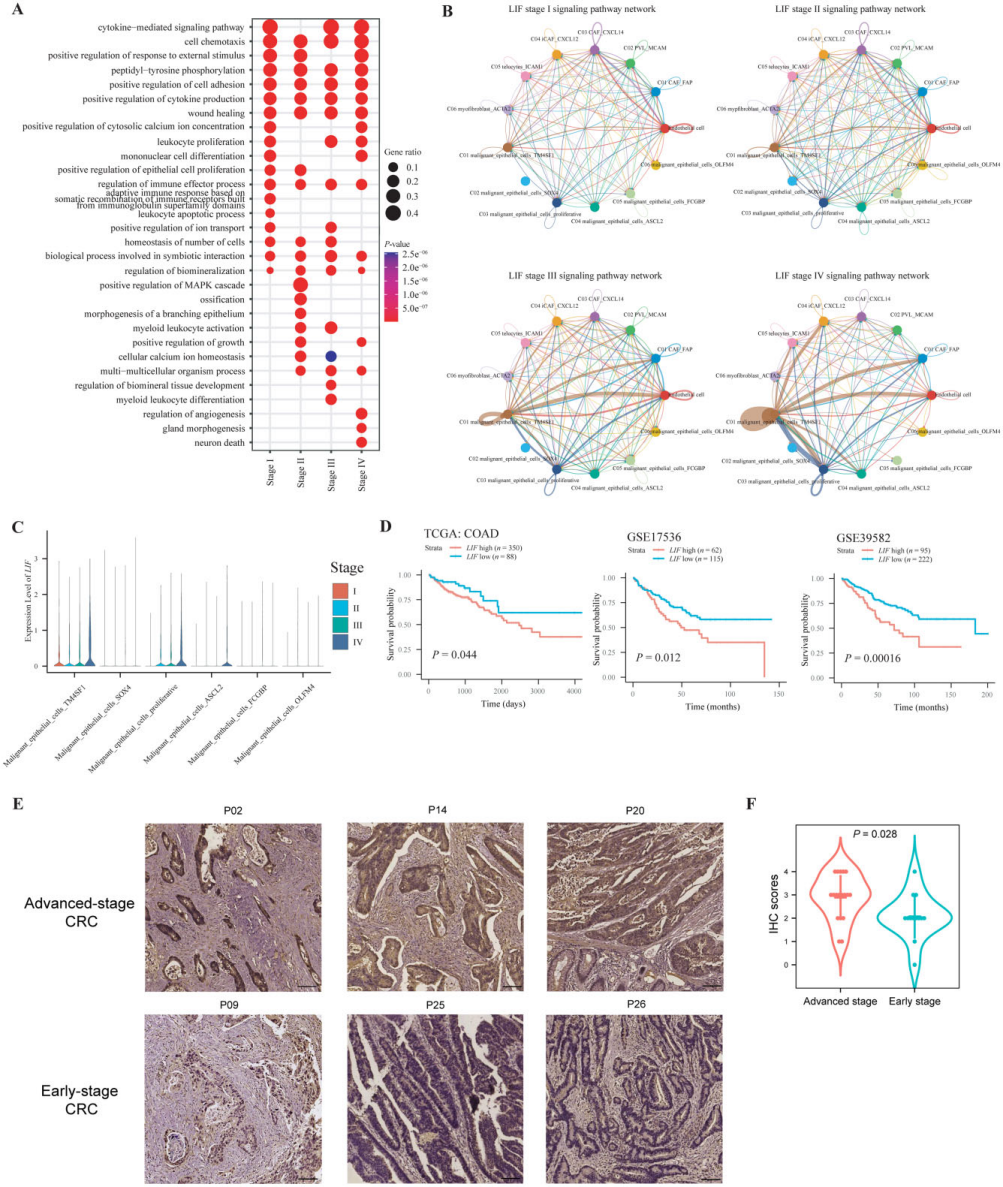
Inferring regulatory hubs in CRC tissues with different stages. (A) GO terms of enriched ligand receptors for each stage of CRC. (B) LIFR signaling pathway networks between malignant epithelial cells and stromal cells are shown in circos plots for stage I–IV CRC. Edge width represents the communication probability. (C) Violin plot depicting the expression of LIF for malignant epithelial cell types in each stage of CRC. (D) Kaplan–Meier curves illustrating the OS for patients from TCGA–COAD, GSE17536, and GSE39584 stratified by low and high expression of LIF. A two-sided log-rank test was performed. (E) Representative images of IHC staining for LIF protein in tumor tissues from patients with early-stage and advanced-stage CRC. (F) IHC analysis of LIF expression. *n *=* *13 for early-stage CRC tissues; *n *=* *18 for advanced CRC tissues; *t*-test was performed to assess the significance. CRC, colorectal cancer; GO, gene ontology; CAF, cancer-associated fibroblast; iCAF, inflammatory CAF; PVL, perivascular-like; OS, overall survival; TCGA, The Cancer Genome Atlas; COAD, colon adenocarcinoma; IHC, immunohistochemistry.

### Altered cancer-associated regulatory hubs were observed in different TNM stages of CRC

Since we have unveiled the dynamic heterogeneity of epithelial cells, stromal cells, and immune cells during CRC progression, we considered that intercellular communications between tumor cells and surrounding stromal as well as immune cells not only engage in immunoregulation, but also exert effects on tumor behaviors, such as proliferation, metastasis, and drug resistance. We have found the colocalization of macrophages, DC, T cells, and tumor cells in TME. However, the alteration of intercellular interaction in each stage of CRC has not been elucidated. Here, we defined communication networks comprising ligands and receptors that could significantly affect the biological behaviors of tumor cells as cancer-associated regulatory hubs. These regulatory hubs contributed to the characteristics of tumor cells and TME that could be correlated with prognosis.

To infer regulatory hubs, we analysed the enriched ligands and receptors in each stage by comparing their differential expression in sequenced stages of all cells. We found that considerable interactions were enriched in specific stages (Supplementary Figure 9A–C). For example, pathways of VEGF, non-canonical WNT, and pleiotrophin, which were important for the growth of tumor cells, were enriched in stage I CRC. On the contrary, pathways of TNF, IL1, IL2, IL4, and IL10, which were associated with immunoregulation, were upregulated in stage IV CRC (Supplementary Figure 9D). These results indicated that cancer-associated regulatory hubs existed and were altered during tumorigenesis. We further analysed the enriched pathways of upregulated and downregulated ligands or receptors during tumorigenesis. GO analysis indicated that several pathways were commonly enriched in stage I–IV CRC, such as cell chemotaxis, cytokine production, and cell adhesion (Figure 8A). However, some pathways were enriched in specific stages of CRC, such as the leukocyte apoptotic process in stage I CRC, positive regulation of the MAPK cascade in stage II CRC, the myeloid leukocyte differentiation pathway in stage III CRC, and the regulation of angiogenesis and gland morphogenesis in stage IV CRC (Figure 8A). Therefore, cancer-associated regulatory hubs were altered in specific stages of CRC and these ligands or receptors could be potential therapeutic targets.

To discover therapeutic targets from cancer-associated hubs, we focused on pathways with increased activity as CRC evolved. As a result, we identified the *LIF–LIFR* interaction was upregulated during CRC progression (Figure 8B). We analysed the intercellular networks about *LIF*/*LIFR* and found that interactions occurred frequently between TM4SF1^+^ tumor cells and proliferative tumor cells, indicating that LIF were mainly secreted from TM4SF1^+^ or proliferative tumor cells exerted an effect on other cell types (Figure 8B). One the other hand, *LIF–LIFR* interaction also occurred between tumor cells and stromal cell types such as CAF and endothelial cells. Expression of *LIF* was higher in TM4SF1^+^ tumor cells and proliferative tumor cells, particularly in advanced CRC tissues (Figure 8C). These results demonstrated that *LIF* was upregulated in tumor cells in advanced CRC. Moreover, a higher expression of *LIF* was associated with worse prognosis (Figure 8D). We further validated the expression of LIF in tumor tissues, which demonstrated that the expression of LIF in tumor cells was higher in patients with advanced-stage CRC than in those with early-stage CRC (Figure 8E and F). Taken together, our results indicated that cancer-associated regulatory hubs could represent intrinsic characteristics of each stage of CRC and LIF–LIFR interaction was discovered to be screwed in advanced CRC.

## Discussion

TME of CRC has been fully characterized by using scRNA-seq. For example, CMS phenotyping, genetic alteration, and infiltration of immune and stromal cells were unveiled at single-cell resolution [25, 30]. However, the alteration of tumor heterogeneity during CRC progression has not been elucidated [24, 25]. This study demonstrated dynamic features including the proportion, function, and lineage differentiation of epithelial cells, stromal cells, as well as immune cells in TME by integrating scRNA-seq and ST-seq data. We showed that TM4SF1 as well as LIF were upregulated as CRC evolved and contributed to a lower OS rate.

In this study, based on their expression on stem cell-, proliferation-, and epithelial lineage-associated genes, malignant epithelial cells were clustered into six subtypes by using differential expression analysis, when fewer and more differentiated tumor cells were ordered by differential trajectory analysis. Stem-cell-associated genes were found to be co-expressed with genes in the Wnt and Bmp signaling pathway such as WNT7A, BMP2, and BMP4. Expression of proliferative genes and folate metabolism for purine synthesis such as MTHFD1, MTHFD2, TYMS, and SHMT2 were shown to be concordant. Lineage markers expressed in more differentiated malignant cells included *CLCA1* for immature goblet cells; *MUC1*, *FCGBP*, and *SPINK4* for mature goblet cells; and *CHGA* for enteroendocrine cells. These marker genes were supported by the literature [31, 32]. The differentiation imputed by transcription was not the same as the morphology in the pathology, since poorly, moderately or well-differentiated CRC according to pathology reports could also consist of various proportions of these six malignant epithelial cell types, indicating the presence of tumor heterogeneity. Notably, the tumor differentiation grade was significantly associated with the stage at which a low grade was proved to be associated with an advanced stage [33]. Our results of differential abundance analysis also showed that stage IV CRC comprised more poorly differentiated malignant epithelial cells that were defined by transcriptome. On the other hand, recently it was reported that metastasis could occur when the primary tumor was a small mass in patients with CRC [34]. Consistently with this, our study unveiled that less-differentiated malignant epithelial cells also occurred in early-stage CRC, suggesting the potential for progression to an advanced stage. Based on differentiation-related genes, seven gene clusters were found to define the transcriptional profile of tumor cells during tumor progression. These gene clusters allowed us to elucidate the alteration of tumor cells during tumor growth and progression. GO enrichment analysis identified that absorptive and secretory cell lineage-associated pathways were enriched in more differentiated tumor cells when pathways responding to low-oxygen conditions as well as the regulation of cell adhesion were enriched in less-differentiated counterparts. Among all differentiation-related genes, *TM4SF1* was highly expressed in the cluster enriched in stage IV CRC. In particular, TM4SF1 was previously reported as one of the markers of cancer stem cells, suggesting that TM4SF1^+^ malignant epithelial cells might have the potential to differentiate to other malignant epithelial cell subtypes [35]. In addition, qRT–PCR validated the increased expression of *TM4SF1* in CRC tissues.

CAF plays an important role in tumor immune evasion and progression. CAF could prevent the infiltration of immune cells, especially cytotoxicity T cells, and contribute to poor prognosis [36]. On the other hand, CAF also exerted their effects on tumor cells by secreting growth factors, cytokines, and exosomes to alter the phenotype of the tumor and promote progression [37–39]. However, how CAF originated from intestinal stromal cells and their remodeling process during tumor progression were not elucidated. As shown by the differentiation trajectory, MCAM^+^ PVL could differentiate into CXCL14^+^ CAF, CXCL12^+^ iCAF, and ACTA2^+^ myofibroblasts. These results were similar to those of a recent study which suggested that ACTA2^+^ CAF emerged through proliferation from intestinal peri-cryptal cells expressing *MCAM* [40]. However, besides myofibroblasts, we further identified the other two branches of differentiation for CAF and revealed that the number of CXCL12^+^ CAF was increased during tumor progression and became more fibrotic based on the expression of transcription factor *JUN*.

The state of immune cells was also remodeled during CRC progression. For example, CD4^+^ Treg, CD4^+^ Trm, IgA^+^ plasma cells, C1QC^+^ macrophages, and mast cells were enriched in advanced CRC. Analysis of the differentiation trajectory and metabolism indicated that terminal states of T, B, and myeloid cells presented the highest metabolism activity. As for T cells, CD4^+^ or CD8^+^ T cells showed a positive correlation between the TCR signal and metabolism activity. Furthermore, for CD8^+^ T cells, exhaustion scores were also positively correlated with metabolic scores and cytotoxic scores. These results showed that the intensity of the dysfunctional signature was positively associated with antitumor immunity for CD8^+^ T cells, which was also reported in melanoma [41]. Tumors with significant T-cell infiltration were associated with better immune checkpoint inhibitor efficacy [42]. To have a deeper understanding of the heterogeneity of immune checkpoints expressed in T cells, we inferred the trend of immune checkpoint expressions for each branch. The different expressions of known immune checkpoints such as *PDCD1*, *CTLA2*, *TIGIT*, *TOX*, *HAVCR2*, and *LAG3* in the lineages of T cells indicated their specific functions in T-cell subtypes and immune checkpoint inhibitors could exert variable effects on T-cell lineages.

The components and colocalization of cell types in CRC were complicated and intercellular communications were other vital factors that could affect TME, making it inflammatory or immunosuppressive. It should be taken into consideration that intercellular communication networks could be altered in different stages of CRC. Inspired by weighted gene co-expression network analysis that demonstrated the correlation of genes in bulk RNA-seq data [43], we defined communication networks that could significantly affect biological behaviors of tumors as cancer-associated regulatory hubs imputed by scRNA-seq data. These regulatory hubs determined the regulation of antitumor immunity as well as tumor growth, progression, and metastasis. Unlike weighted correlation network analysis, which took all gene hubs into consideration, including both intracellular signal pathways and intercellular communications, our analytical pipeline only focused on cell–cell interactions mainly comprising ligands and receptors. The advantages of our method were the ability to accurately find the key regulatory hubs and the associated cell types. As a result, we successfully discovered some regulatory hubs in each stage of CRC. The cascade of these regulatory hubs was associated with CRC progression. For example, the leukocyte apoptotic process, including IL10, PDCD1, and IDO, could facilitate the initiation of CRC by contributing to the immunosuppressive TME. The MAPK cascade, which is a key factor in evading apoptosis, regulating chemotherapy resistance, and promoting metastasis, was enriched in stage II CRC [44, 45]. And regulation of angiogenesis played an important role in stage IV CRC. Next, we focused on regulatory hubs that were upregulated in advanced CRC. *LIF–LIFR* interaction was found to be upregulated when CRC evolved and predominantly occurred in TM4SF1^+^ or MKI67^+^ malignant epithelial cells. LIF was overexpressed in many solid tumors, which could bind with LIFR and activate oncogenic signaling pathways including JAK/STAT3, MAPK, AKT, and mTOR [46]. A previous study reported that LIF negatively regulated tumor-suppressor p53 through STAT3/ID1/MDM2 signaling in CRC [47]. We utilized immunohistochemistry (IHC) staining to verify the different expression of LIF in CRC. LIF was mainly expressed in tumor cells and increased in advanced tumors. Moreover, high expression of LIF in patients with CRC was associated with bad prognosis. These results proved that the LIF–LIFR was an important cancer-associated regulatory hub in advanced CRC.

Our study has provided a new analytic strategy to identify the dynamic heterogeneity of CRC during tumorigenesis. However, there are several limitations to our study. First, the characteristics of the immune environment in different parts of the large bowel, such as the colon and rectum, may be different, so it is optimal to analyse the dynamic landscape of CRC with different stages by taking the location of the tumor into consideration. However, this is limited by the availability of the current data, which can be solved with the accumulation of more data of scRNA-seq on CRC. Second, data on the spatial transcription of CRC are only available in a small number of patients, which prevents us from performing a meticulous analysis of the spatial features of CRC.

In conclusion, our study unveiled the dynamics of heterogeneity during CRC progression, including cell-type proportion, function, and lineages, which contributed to the alteration of cancer-associated regulatory hubs. Particularly, we found that TM4SF1 and LIF might serve as tumor progression markers in patients with CRC.

## Supplementary Material

goad034_Supplementary_Data
