# Supplementary material for: Dynamic heterogeneity of colorectal cancer during progression revealed clinical risk-associated cell types and regulations in single-cell resolution and spatial context
Source: Gastroenterol Rep (Oxf). 2023 Jun 24;11:goad034. doi: 10.1093/gastro/goad034 (PMC10290555; doi:10.1093/gastro/goad034)
Supplement: goad034_Supplementary_Data [file goad034_supplementary_data.zip › Supplementary_figure.pdf]

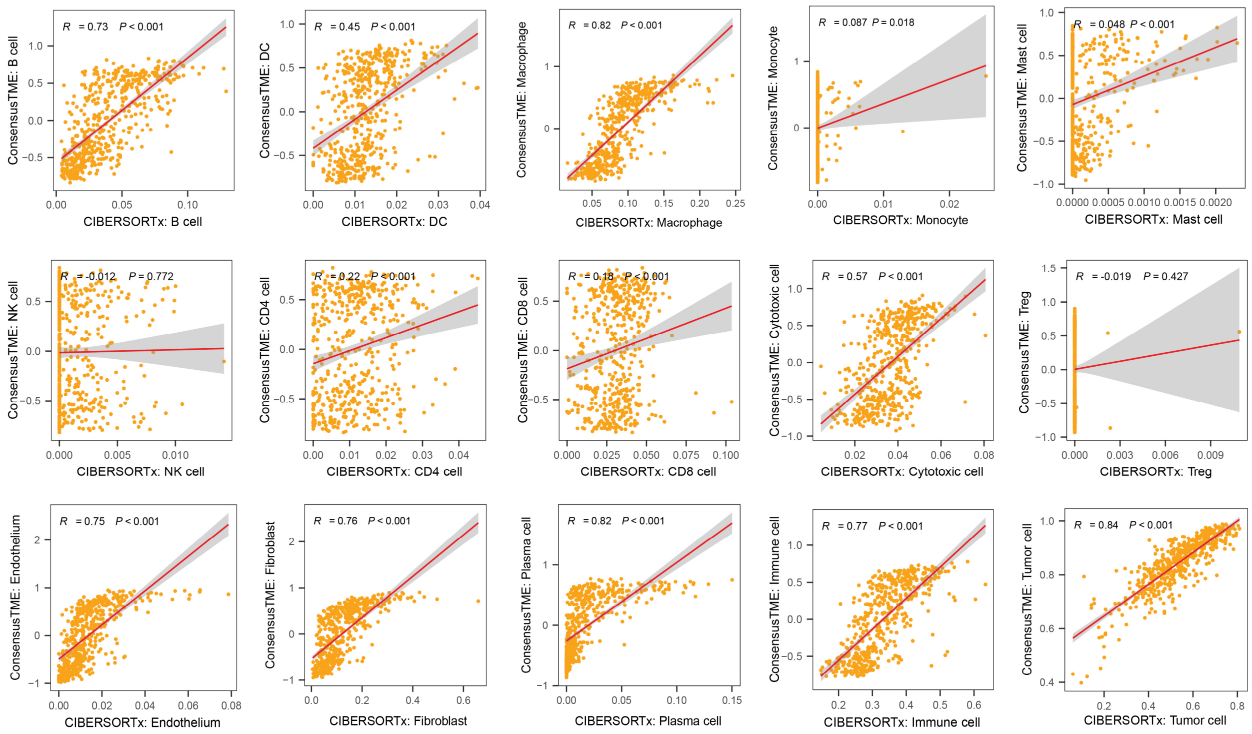

Supplementary Figure 1. The Pearson correlation between the fraction of cell types analyzed by CIBERSORTx and corresponding enrichment score generated by R package consensusTME using GSVA for TCGA-COAD RNA-seq dataset. The *P*-value for Pearson's correlation coefficients was calculated using a t-distribution with *n* - 2 degrees. DC, dendritic cell; GSVA, gene set variation analysis.

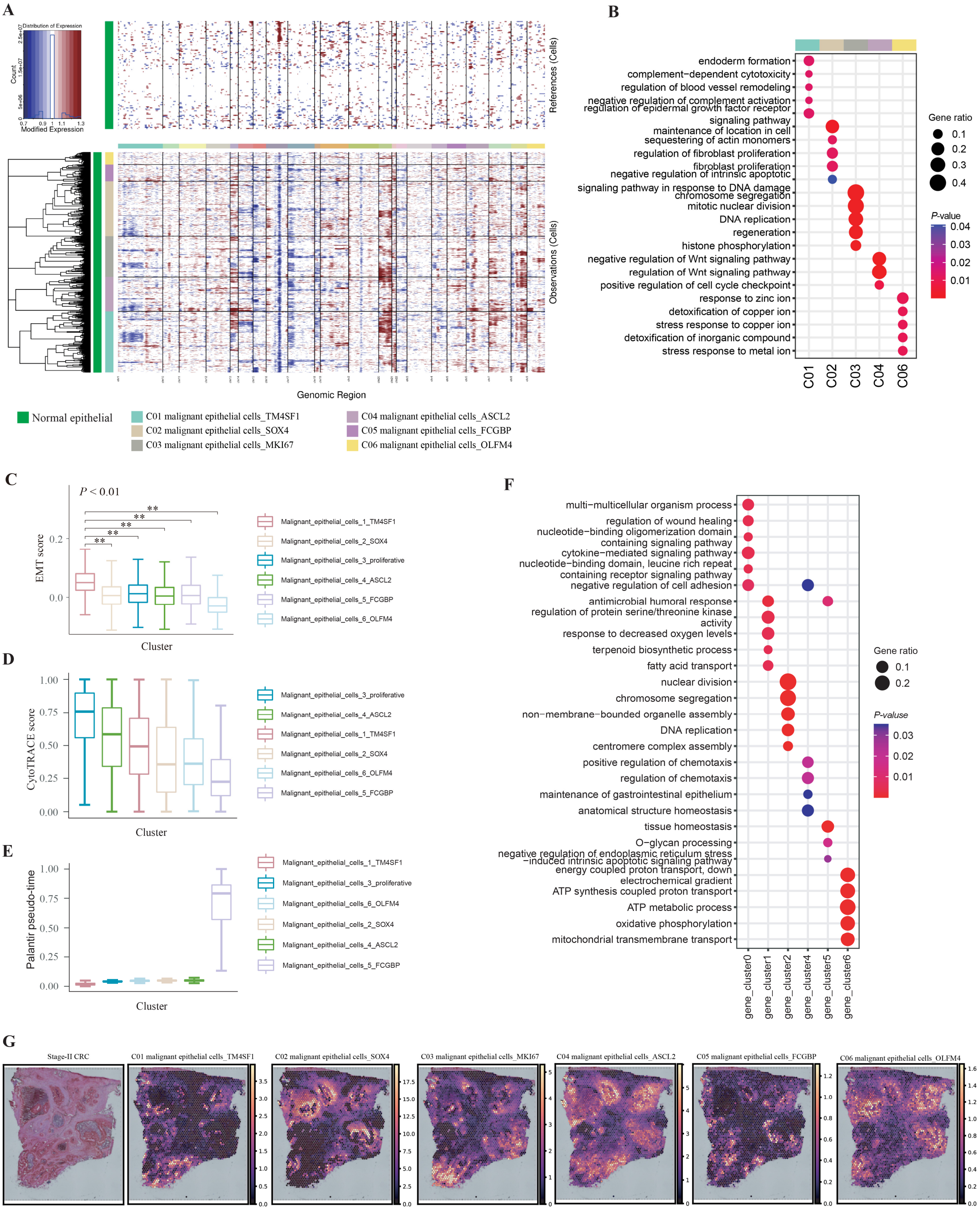
A
B
C
D
E
F
G
Distribution of Expression
Count
Modified Expression
References (Cells)
Observations (Cells)
Genomic Region
Normal epithelial
C01 malignant epithelial cells_TM4SF1
C02 malignant epithelial cells_SOX4
C03 malignant epithelial cells_MKI67
C04 malignant epithelial cells_ASCL2
C05 malignant epithelial cells_FCGBP
C06 malignant epithelial cells_OLFM4
endoderm formation
complement−dependent cytotoxicity
regulation of blood vessel remodeling
negative regulation of complement activation
regulation of epidermal growth factor receptor signaling pathway
maintenance of location in cell
sequestering of actin monomers
regulation of fibroblast proliferation
fibroblast proliferation
negative regulation of intrinsic apoptotic signaling pathway in response to DNA damage
chromosome segregation
mitotic nuclear division
DNA replication
regeneration
histone phosphorylation
negative regulation of Wnt signaling pathway
regulation of Wnt signaling pathway
positive regulation of cell cycle checkpoint
response to zinc ion
detoxification of copper ion
stress response to copper ion
detoxification of inorganic compound
stress response to metal ion
C01
C02
C03
C04
C06
Gene ratio
0.1
0.2
0.3
0.4
P-value
0.04
0.03
0.02
0.01
P < 0.01
EMT score
Cluster
Malignant_epithelial_cells_1_TM4SF1
Malignant_epithelial_cells_2_SOX4
Malignant_epithelial_cells_3_proliferative
Malignant_epithelial_cells_4_ASCL2
Malignant_epithelial_cells_5_FCGBP
Malignant_epithelial_cells_6_OLFM4
CytoTRACE score
Palantir pseudo-time
multi−multicellular organism process
regulation of wound healing
nucleotide−binding oligomerization domain containing signaling pathway
cytokine−mediated signaling pathway
nucleotide−binding domain, leucine rich repeat containing receptor signaling pathway
negative regulation of cell adhesion
antimicrobial humoral response
regulation of protein serine/threonine kinase activity
response to decreased oxygen levels
terpenoid biosynthetic process
fatty acid transport
nuclear division
chromosome segregation
non−membrane−bounded organelle assembly
DNA replication
centromere complex assembly
positive regulation of chemotaxis
regulation of chemotaxis
maintenance of gastrointestinal epithelium
anatomical structure homeostasis
tissue homeostasis
O−glycan processing
negative regulation of endoplasmic reticulum stress −induced intrinsic apoptotic signaling pathway
energy coupled proton transport, down electrochemical gradient
ATP synthesis coupled proton transport
ATP metabolic process
oxidative phosphorylation
mitochondrial transmembrane transport
gene_cluster0
gene_cluster1
gene_cluster2
gene_cluster4
gene_cluster5
gene_cluster6
P-valuse
Stage-II CRC
C01 malignant epithelial cells_TM4SF1
C02 malignant epithelial cells_SOX4
C03 malignant epithelial cells_MKI67
C04 malignant epithelial cells_ASCL2
C05 malignant epithelial cells_FCGBP
C06 malignant epithelial cells_OLFM4

Supplementary Figure 2. (A) Heatmap showed large-scale copy number variation profile of each malignant epithelial cell type. Red and blue colors represent high and low copy number variation level, respectively. Normal epithelial cells were selected as reference. **(B)** GO analysis of upregulated genes of each malignant epithelial cluster. **(C)** Box plot showed EMT scores of each malignant epithelial cell types. Data were shown by median with interquartile range, *P*-value was calculated by one-way ANOVA and Tukey's post hoc test, $^{*}P < 0.05$; $^{**}P < 0.01$. **(D)** Box plot demonstrated CytoTRACE scores of malignant epithelial cell types. **(E)** Box plot illustrated the psuedo-time order of malignant epithelial cell types imputed by Palantir. Data were shown by median with interquartile range (C-D). **(F)** GO analysis results of genes in each gene cluster computed by Palantir which were also overlapped with upregulated genes of all malignant epithelial cell clusters. **(G)** Spatial abundance of malignant epithelial clusters estimated by cell2location was shown on the slice of a stage-II CRC with the matched image of H & E staining. GO, gene ontology; EMT, epithelial-mesenchymal transition; H & E, hematoxylin and eosin; CRC, colorectal cancer.

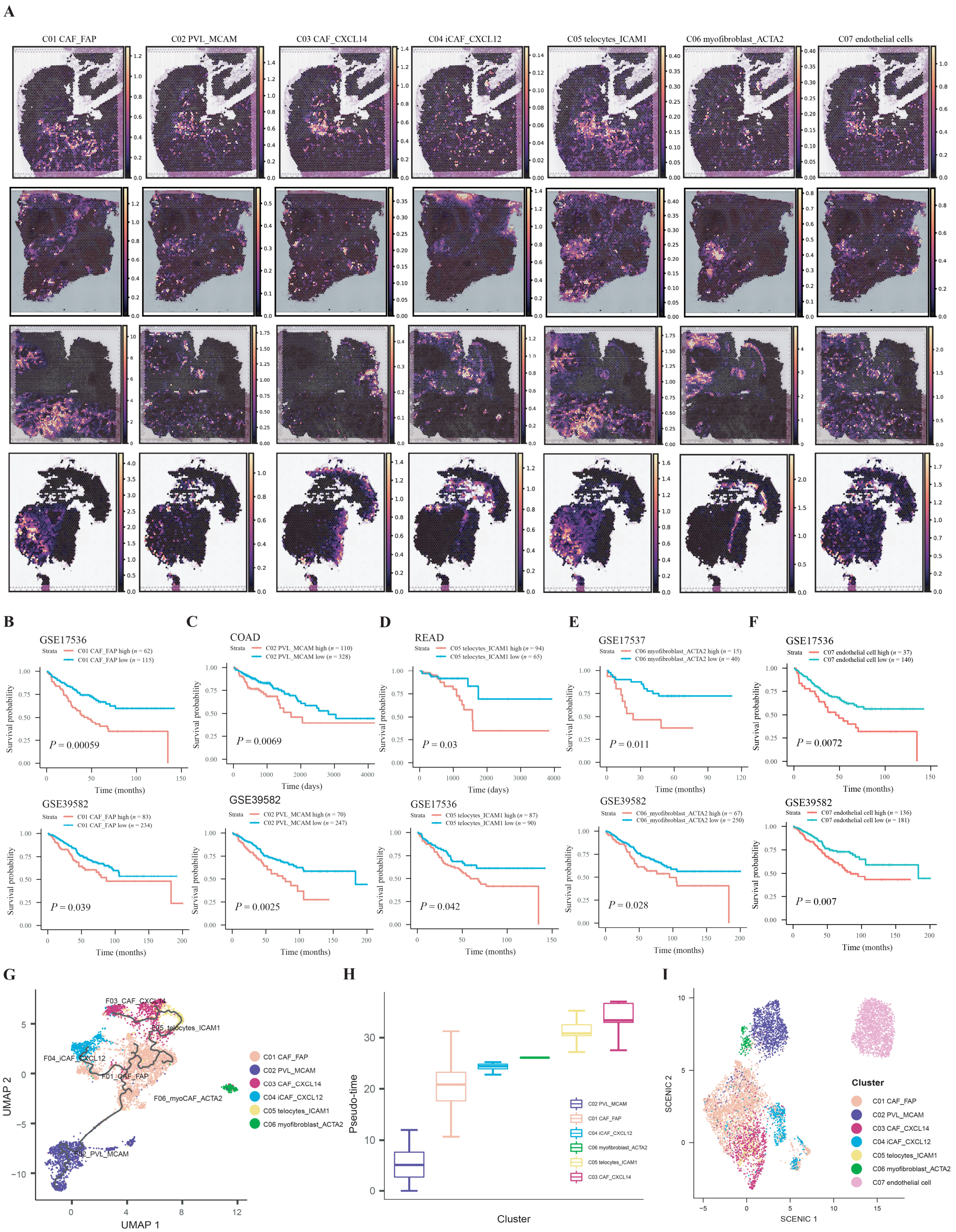
A
C01 CAF_FAP
C02 PVL_MCAM
C03 CAF_CXCL14
C04 iCAF_CXCL12
C05 telocytes_ICAM1
C06 myofibroblast_ACTA2
C07 endothelial cells
B
GSE17536
Strata
C01 CAF_FAP high (n = 62)
C01 CAF_FAP low (n = 115)
P = 0.00059
Survival probability
Time (months)
C
COAD
C02 PVL_MCAM high (n = 110)
C02 PVL_MCAM low (n = 328)
P = 0.0069
Time (days)
D
READ
C05 telocytes_ICAM1 high (n = 94)
C05 telocytes_ICAM1 low (n = 65)
P = 0.03
Time (days)
E
GSE17537
C06 myofibroblast_ACTA2 high (n = 15)
C06 myofibroblast_ACTA2 low (n = 40)
P = 0.011
Time (months)
F
GSE17536
C07 endothelial cell high (n = 37)
C07 endothelial cell low (n = 140)
P = 0.0072
Time (months)
GSE39582
C01 CAF_FAP high (n = 83)
C01 CAF_FAP low (n = 234)
P = 0.039
GSE39582
C02 PVL_MCAM high (n = 70)
C02 PVL_MCAM low (n = 247)
P = 0.0025
GSE17536
C05 telocytes_ICAM1 high (n = 87)
C05 telocytes_ICAM1 low (n = 90)
P = 0.042
GSE39582
C06_myofibroblast_ACTA2 high (n = 67)
C06_myofibroblast_ACTA2 low (n = 250)
P = 0.028
GSE39582
C07 endothelial cell high (n = 136)
C07 endothelial cell low (n = 181)
P = 0.007
G
F03_CAF_CXCL14
F05_telocytes_ICAM1
F04_iCAF_CXCL12
F01_CAF_FAP
F06_myoCAF_ACTA2
F02_PVL_MCAM
UMAP 1
UMAP 2
C01 CAF_FAP
C02 PVL_MCAM
C03 CAF_CXCL14
C04 iCAF_CXCL12
C05 telocytes_ICAM1
C06 myofibroblast_ACTA2
H
Pseudo-time
Cluster
C02 PVL_MCAM
C01 CAF_FAP
C04 iCAF_CXCL12
C06 myofibroblast_ACTA2
C05 telocytes_ICAM1
C03 CAF_CXCL14
I
SCENIC 1
SCENIC 2
Cluster
C01 CAF_FAP
C02 PVL_MCAM
C03 CAF_CXCL14
C04 iCAF_CXCL12
C05 telocytes_ICAM1
C06 myofibroblast_ACTA2
C07 endothelial cell

Supplementary Figure 3. (A) Spatial abundance of stromal cell types estimated by cell2location. Slices of four CRC patients were shown, including a tumor core from stage-IV CRC, a tumor core from stage-II CRC, and two CRC border tissues, from top to bottom. **(B-F)** Overall survival analysis for TCGA-COAD, TCGA-READ, GSE17536, GSE17537, and GSE39582 patients stratified by the infiltration of FAP^{+} CAF (B), $MCAM^{+}$ PVL (C), $ICAM1^{+}$ telocyte (D), $ACTA2^{+}$ myofibroblast (E) and endothelial cells (F) using Kaplan-Meier curves by two-sided log-rank test. **(G)** Differentiation trajectory of MSC analyzed by monocle3 was demonstrated in UMAP plot. **(H)** Box plot of pseudo-time for each stromal cell type analyzed by monocle3. Data were shown by median with interquartile range. **(I)** UMAP plot depicted stromal cells clustered in regulon space (SCENIC analysis). CRC, colorectal cancer; CAF, cancer associated fibroblast; iCAF, inflammatory CAF; PVL, perivascular-like; GO, gene ontology; TCGA, the Cancer Genome Atlas; COAD, colon adenocarcinoma; READ, rectum adenocarcinoma; MSC, mesenchymal stromal cells; UMAP, Uniform Manifold Approximation and Projection.

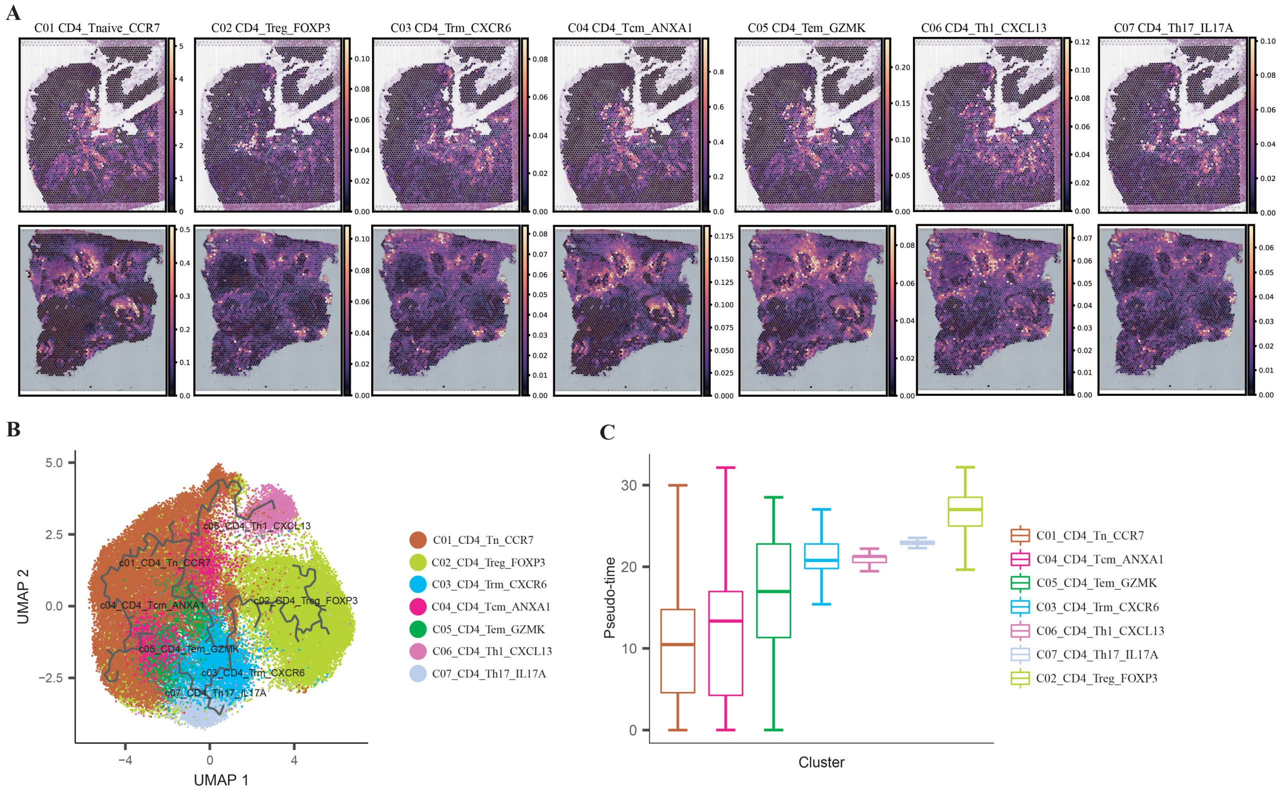

Supplementary Figure 4. **(A)** Spatial abundance of $CD4^+$ T cell types estimated by cell2location from slices of stage-IV CRC tissue (top) and stage-II CRC tissues. **(B)** Differentiation trajectory of $CD4^+$ T cells imputed by monocle3 was shown in UMAP plot. **(C)** Bar plot depicted pseudo-time order of $CD4^+$ T cell type analyzed by monocle3. Data were shown by median with interquartile range. CRC, colorectal cancer; Trm, resident memory T cell; Tcm, central memory T cell; Tem, effector memory T cell; UMAP, Uniform Manifold Approximation and Projection.

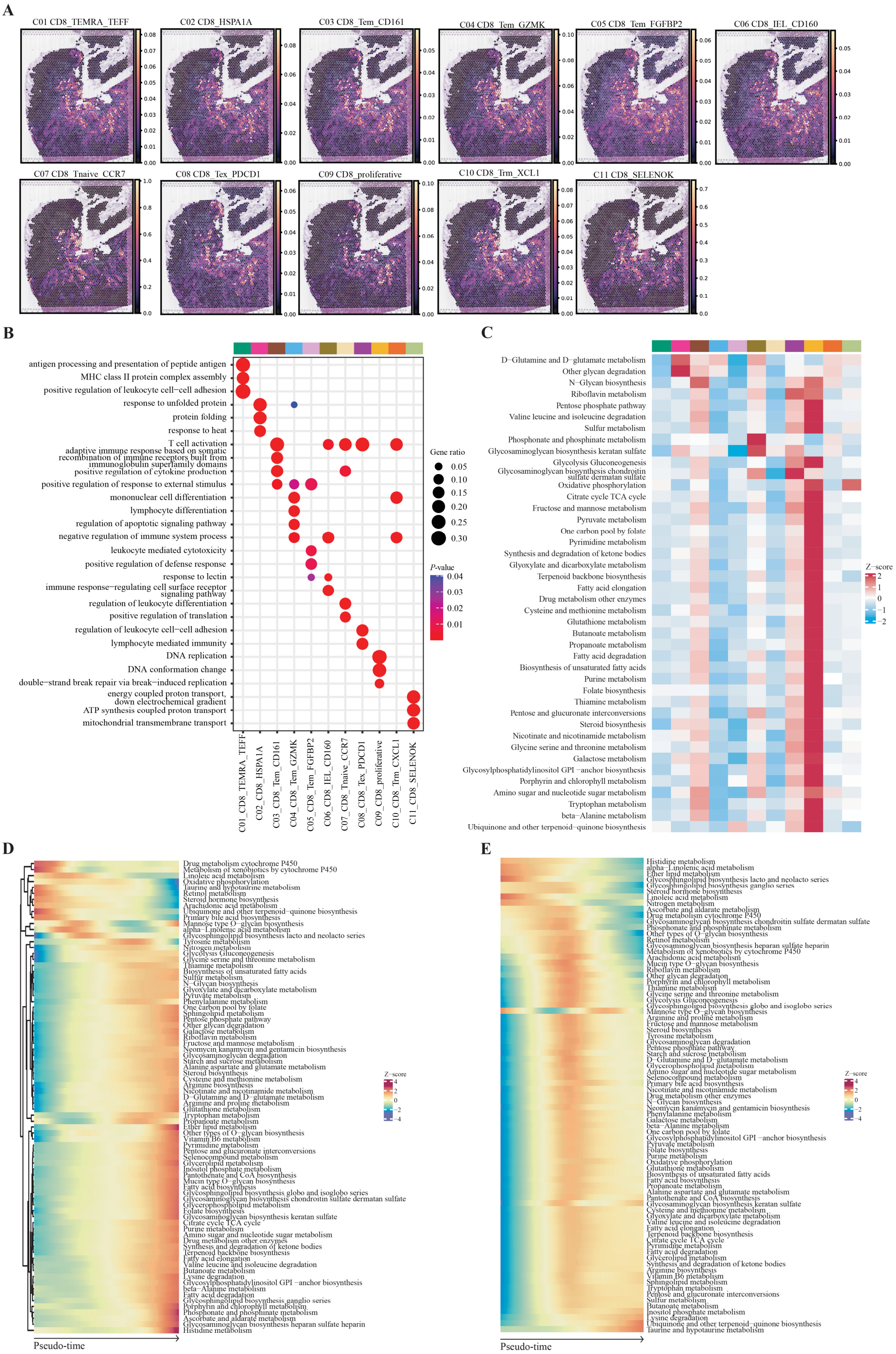
A
C01 CD8_TEMRA_TEFF
C02 CD8_HSPA1A
C03 CD8_Tem_CD161
C04 CD8_Tem_GZMK
C05 CD8_Tem_FGFBP2
C06 CD8_IEL_CD160
C07 CD8_Tnaive_CCR7
C08 CD8_Tex_PDCD1
C09 CD8_proliferative
C10 CD8_Trm_XCL1
C11 CD8_SELENOK
B
antigen processing and presentation of peptide antigen
MHC class II protein complex assembly
positive regulation of leukocyte cell−cell adhesion
response to unfolded protein
protein folding
response to heat
T cell activation
adaptive immune response based on somatic recombination of immune receptors built from immunoglobulin superfamily domains
positive regulation of cytokine production
positive regulation of response to external stimulus
mononuclear cell differentiation
lymphocyte differentiation
regulation of apoptotic signaling pathway
negative regulation of immune system process
leukocyte mediated cytotoxicity
positive regulation of defense response
response to lectin
immune response−regulating cell surface receptor signaling pathway
regulation of leukocyte differentiation
positive regulation of translation
regulation of leukocyte cell−cell adhesion
lymphocyte mediated immunity
DNA replication
DNA conformation change
double−strand break repair via break−induced replication
energy coupled proton transport, down electrochemical gradient
ATP synthesis coupled proton transport
mitochondrial transmembrane transport
C01_CD8_TEMRA_TEFF
C02_CD8_HSPA1A
C03_CD8_Tem_CD161
C04_CD8_Tem_GZMK
C05_CD8_Tem_FGFBP2
C06_CD8_IEL_CD160
C07_CD8_Tnaive_CCR7
C08_CD8_Tex_PDCD1
C09_CD8_proliferative
C10_CD8_Trm_XCL1
C11_CD8_SELENOK
Gene ratio
0.05
0.10
0.15
0.20
0.25
0.30
P-value
0.04
0.03
0.02
0.01
C
D−Glutamine and D−glutamate metabolism
Other glycan degradation
N−Glycan biosynthesis
Riboflavin metabolism
Pentose phosphate pathway
Valine leucine and isoleucine degradation
Sulfur metabolism
Phosphonate and phosphinate metabolism
Glycosaminoglycan biosynthesis keratan sulfate
Glycolysis Gluconeogenesis
Glycosaminoglycan biosynthesis chondroitin sulfate dermatan sulfate
Oxidative phosphorylation
Citrate cycle TCA cycle
Fructose and mannose metabolism
Pyruvate metabolism
One carbon pool by folate
Pyrimidine metabolism
Synthesis and degradation of ketone bodies
Glyoxylate and dicarboxylate metabolism
Terpenoid backbone biosynthesis
Fatty acid elongation
Drug metabolism other enzymes
Cysteine and methionine metabolism
Glutathione metabolism
Butanoate metabolism
Propanoate metabolism
Fatty acid degradation
Biosynthesis of unsaturated fatty acids
Purine metabolism
Folate biosynthesis
Thiamine metabolism
Pentose and glucuronate interconversions
Steroid biosynthesis
Nicotinate and nicotinamide metabolism
Glycine serine and threonine metabolism
Galactose metabolism
Glycosylphosphatidylinositol GPI −anchor biosynthesis
Porphyrin and chlorophyll metabolism
Amino sugar and nucleotide sugar metabolism
Tryptophan metabolism
beta−Alanine metabolism
Ubiquinone and other terpenoid−quinone biosynthesis
Z−score
2
1
0
−1
−2
D
Pseudo-time
Z−score
E
Pseudo-time
Z−score

Supplementary Figure 5. **(A)** Spatial abundance of $CD8^+$ T cell types in spatial transcription estimated by cell2location from a slice of stage-IV CRC tissue. **(B)** GO analysis of upregulated genes for each $CD8^+$ T cell type. **(C)** Heatmap showed the activity of metabolic pathways for each $CD8^+$ T cell type. The top color bars indicated $CD8^+$ T cell clusters as in (B). **(D)** Heatmap illustrated the trend of metabolism activity as regards to the differentiation procedure from naïve $CD8^+$ T cell to $CD160^+$ IEL. **(E)** Heatmap demonstrated the trend of metabolism activity as regards to differentiation procedure from naïve $CD8^+$ T cell to proliferative $CD8^+$ T cell. CRC, colorectal cancer; Trm, resident memory T cell; Tem, effector memory T cell; TEMRA/TEFF, recently activated effector memory or effector T cell; IEL, intraepithelial lymphocyte; Tex, exhausted T cell; GO, gene ontology.

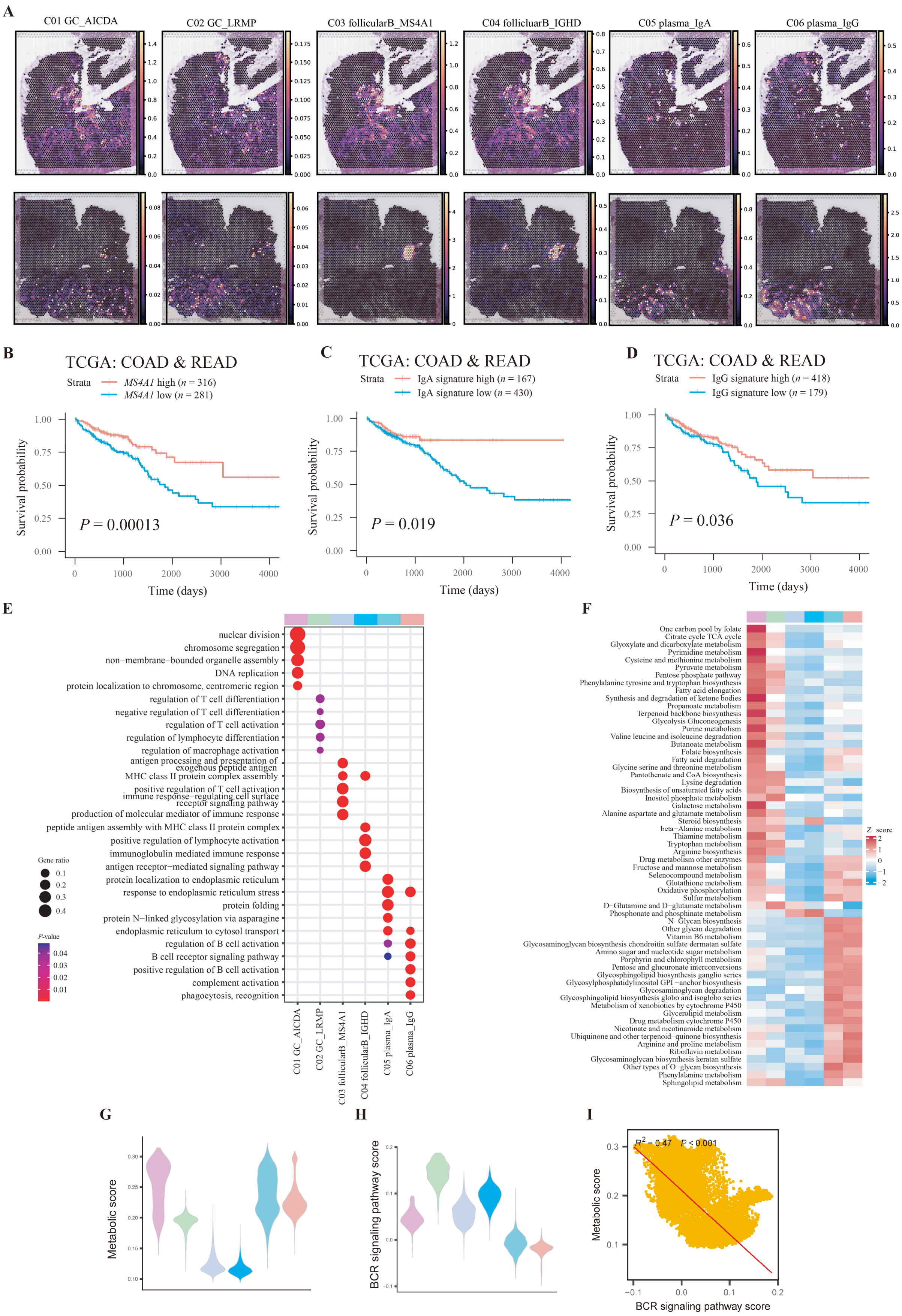
A
C01 GC_AICDA
C02 GC_LRMP
C03 follicularB_MS4A1
C04 folliclularB_IGHD
C05 plasma_IgA
C06 plasma_IgG
B
TCGA: COAD & READ
Strata
MS4A1 high (n = 316)
MS4A1 low (n = 281)
Survival probability
P = 0.00013
Time (days)
C
TCGA: COAD & READ
Strata
IgA signature high (n = 167)
IgA signature low (n = 430)
Survival probability
P = 0.019
Time (days)
D
TCGA: COAD & READ
Strata
IgG signature high (n = 418)
IgG signature low (n = 179)
Survival probability
P = 0.036
Time (days)
E
nuclear division
chromosome segregation
non−membrane−bounded organelle assembly
DNA replication
protein localization to chromosome, centromeric region
regulation of T cell differentiation
negative regulation of T cell differentiation
regulation of T cell activation
regulation of lymphocyte differentiation
regulation of macrophage activation
antigen processing and presentation of exogenous peptide antigen
MHC class II protein complex assembly
positive regulation of T cell activation
immune response−regulating cell surface receptor signaling pathway
production of molecular mediator of immune response
peptide antigen assembly with MHC class II protein complex
positive regulation of lymphocyte activation
immunoglobulin mediated immune response
antigen receptor−mediated signaling pathway
protein localization to endoplasmic reticulum
response to endoplasmic reticulum stress
protein folding
protein N−linked glycosylation via asparagine
endoplasmic reticulum to cytosol transport
regulation of B cell activation
B cell receptor signaling pathway
positive regulation of B cell activation
complement activation
phagocytosis, recognition
Gene ratio
P-value
C01 GC_AICDA
C02 GC_LRMP
C03 follicularB_MS4A1
C04 follicularB_IGHD
C05 plasma_IgA
C06 plasma_IgG
F
One carbon pool by folate
Citrate cycle TCA cycle
Glyoxylate and dicarboxylate metabolism
Pyrimidine metabolism
Cysteine and methionine metabolism
Pyruvate metabolism
Pentose phosphate pathway
Phenylalanine tyrosine and tryptophan biosynthesis
Fatty acid elongation
Synthesis and degradation of ketone bodies
Propanoate metabolism
Terpenoid backbone biosynthesis
Glycolysis Gluconeogenesis
Purine metabolism
Valine leucine and isoleucine degradation
Butanoate metabolism
Folate biosynthesis
Fatty acid degradation
Glycine serine and threonine metabolism
Pantothenate and CoA biosynthesis
Lysine degradation
Biosynthesis of unsaturated fatty acids
Inositol phosphate metabolism
Galactose metabolism
Alanine aspartate and glutamate metabolism
Steroid biosynthesis
beta−Alanine metabolism
Thiamine metabolism
Tryptophan metabolism
Arginine biosynthesis
Drug metabolism other enzymes
Fructose and mannose metabolism
Selenocompound metabolism
Glutathione metabolism
Oxidative phosphorylation
Sulfur metabolism
D−Glutamine and D−glutamate metabolism
Phosphonate and phosphinate metabolism
N−Glycan biosynthesis
Other glycan degradation
Vitamin B6 metabolism
Glycosaminoglycan biosynthesis chondroitin sulfate dermatan sulfate
Amino sugar and nucleotide sugar metabolism
Porphyrin and chlorophyll metabolism
Pentose and glucuronate interconversions
Glycosphingolipid biosynthesis ganglio series
Glycosylphosphatidylinositol GPI −anchor biosynthesis
Glycosaminoglycan degradation
Glycosphingolipid biosynthesis globo and isoglobo series
Metabolism of xenobiotics by cytochrome P450
Glycerolipid metabolism
Drug metabolism cytochrome P450
Nicotinate and nicotinamide metabolism
Ubiquinone and other terpenoid−quinone biosynthesis
Arginine and proline metabolism
Riboflavin metabolism
Glycosaminoglycan biosynthesis keratan sulfate
Other types of O−glycan biosynthesis
Phenylalanine metabolism
Sphingolipid metabolism
Z−score
G
Metabolic score
H
BCR signaling pathway score
I
R2 = 0.47 P < 0.001
Metabolic score
BCR signaling pathway score

J

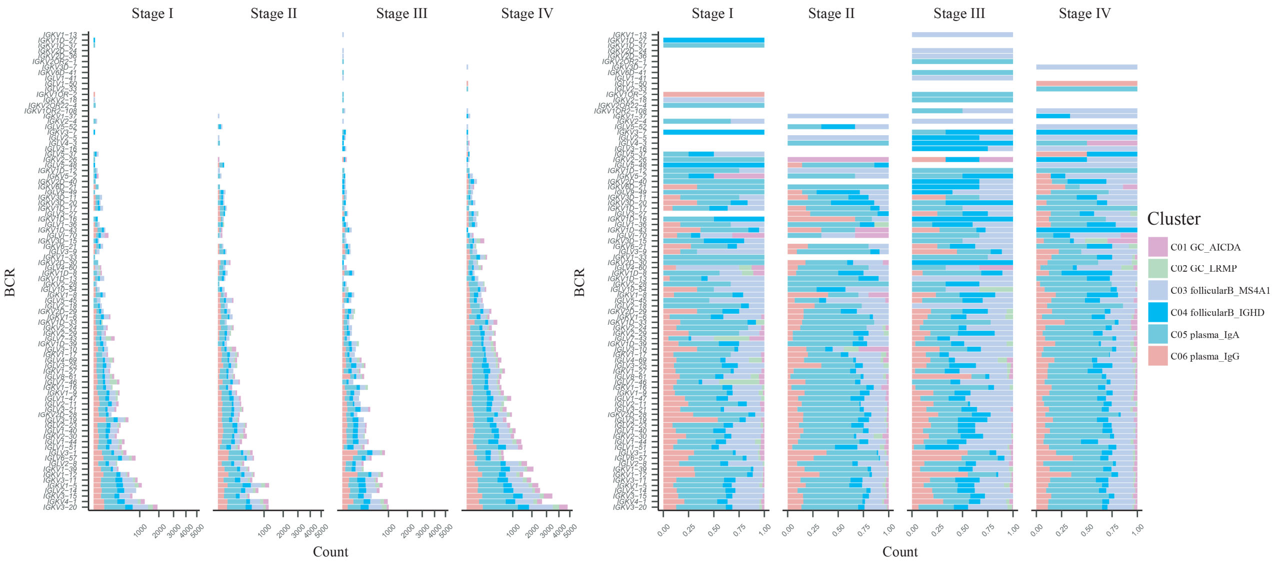

Supplementary Figure 6. (A) Spatial abundance of B cell types analyzed by cell2location from slices of stage-IV CRC (top) or the border of CRC tissues with a TLS (bottom). **(B-D)** Overall survival analysis for TCGA-COAD and TCGA-READ patients stratified by high or low expression of MS4A1 (B), IgA signature (sum of expression of IGHA1 and IGHA2) (C) and IgG signature (sum of expression of IGHG1, IGHG2, IGHG3, IGHG4) (D) using Kaplan-Meier curves by two-sided log-rank test. **(E)** Functional enrichment analysis of upregulated genes in each B cell type. **(F)** Heatmap compared the different activity of metabolic pathways for each B cell type. The top color bars indicated B cell clusters as in (E). **(G)** Violin plot showing the global metabolic activity of B cells estimated by VISION algorithm. The colors indicated B cell clusters as same as in (E). **(H)** Violin plot showing activity of BCR signaling pathway of B cells. The colors indicated B cell clusters as same as in (E). **(I)** Scatter plot demonstrated the correlation between BCR signaling and global metabolic activity in B cells. The error band indicates 95% confidence interval. **(J)** Bar plot showed the distribution of variable regions in BCR light chain for each stage of CRC. CRC, colorectal cancer; TLS, tertiary lymphoid structures; TCGA, the Cancer Genome Atlas; COAD, colon adenocarcinoma; READ, rectum adenocarcinoma; UMAP, Uniform Manifold Approximation and Projection; BCR, B cell receptor.

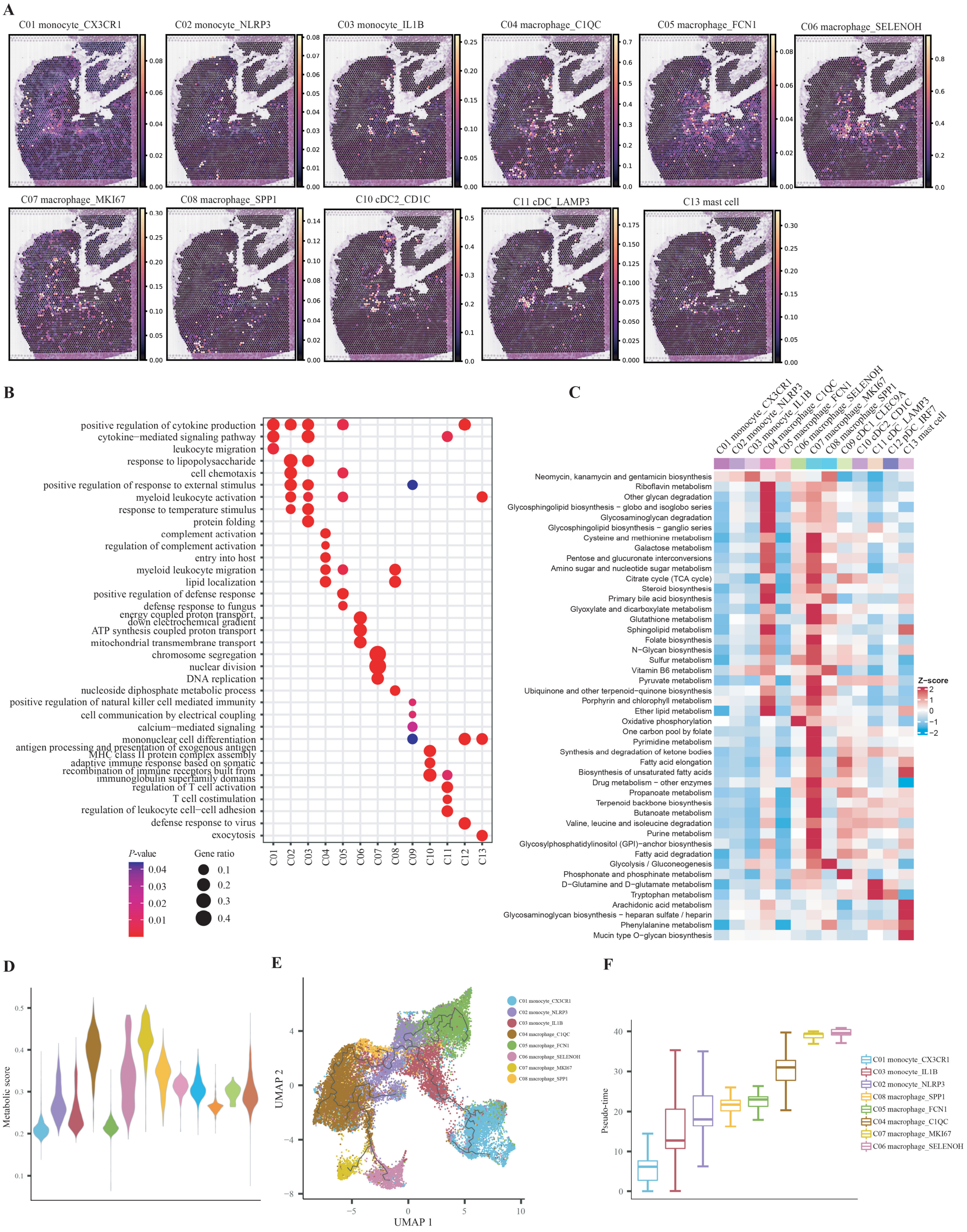

Supplementary Figure 7. (A) Spatial abundance of myeloid cell clusters analyzed by cell2location from a slice of stage-IV CRC tissue. **(B)** GO analysis results of upregulated genes of each myeloid cell

type. **(C)** Heatmap compared the activity of metabolic pathways for each myeloid cell type. **(D)** The global metabolic activity of each myeloid cell type shown in Violin plot. **(E)** Differentiation trajectory of monocytes and macrophages imputed by monocle3 was shown in UMAP plot. **(F)** Bar plot depicted pseudo-time order of monocytes and macrophages analyzed by monocle3. Data were shown by median with interquartile range. CRC, colorectal cancer; cDC, classical dendritic cell; pDC, plasmacytoid dendritic cell; UMAP, Uniform Manifold Approximation and Projection;

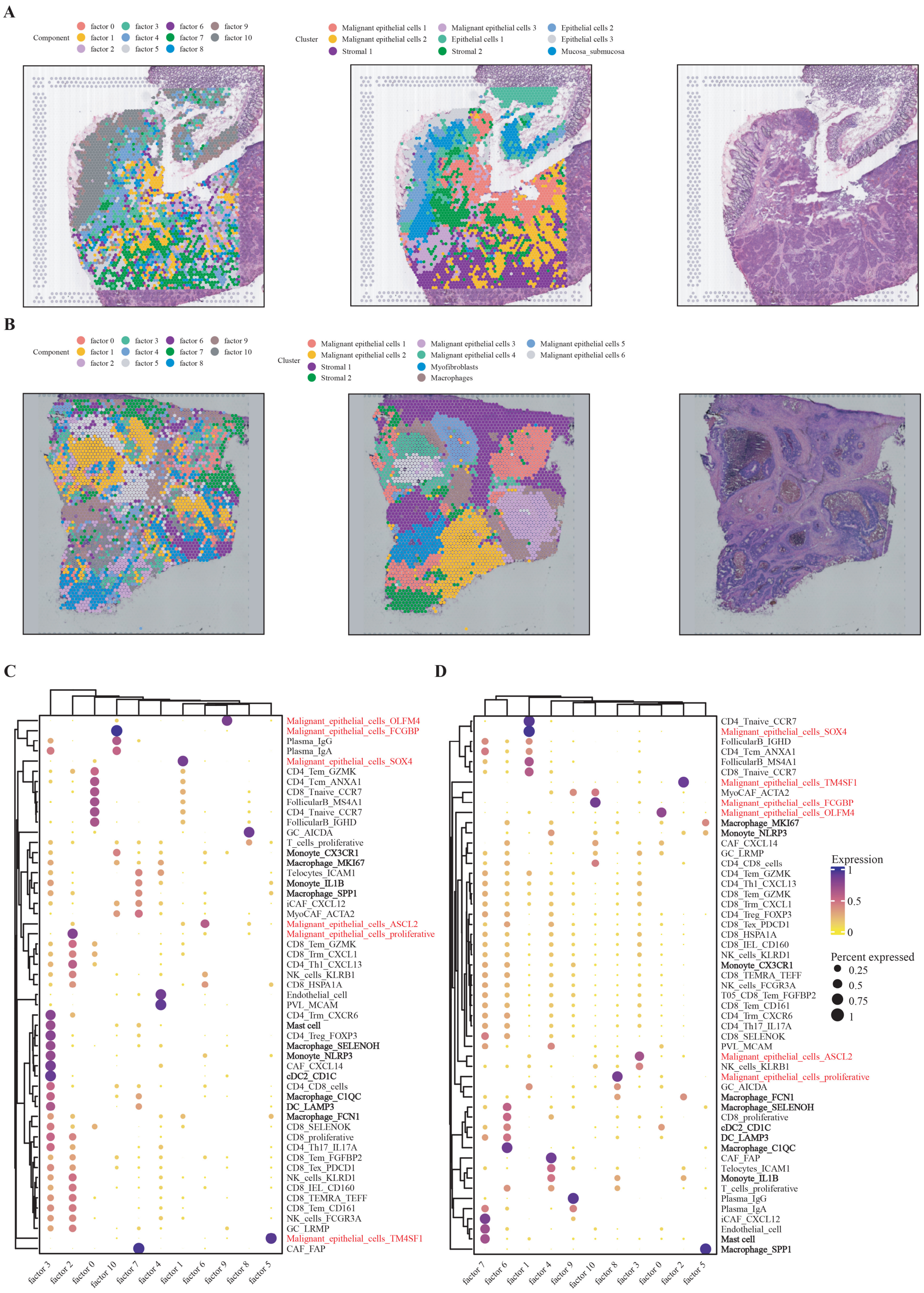
A
Component
factor 0
factor 1
factor 2
factor 3
factor 4
factor 5
factor 6
factor 7
factor 8
factor 9
factor 10
Cluster
Malignant epithelial cells 1
Malignant epithelial cells 2
Stromal 1
Malignant epithelial cells 3
Epithelial cells 1
Stromal 2
Epithelial cells 2
Epithelial cells 3
Mucosa_submucosa
B
Component
factor 0
factor 1
factor 2
factor 3
factor 4
factor 5
factor 6
factor 7
factor 8
factor 9
factor 10
Cluster
Malignant epithelial cells 1
Malignant epithelial cells 2
Stromal 1
Stromal 2
Malignant epithelial cells 3
Malignant epithelial cells 4
Myofibroblasts
Macrophages
Malignant epithelial cells 5
Malignant epithelial cells 6
C
Malignant_epithelial_cells_OLFM4
Malignant_epithelial_cells_FCGBP
Plasma_IgG
Plasma_IgA
Malignant_epithelial_cells_SOX4
CD4_Tem_GZMK
CD4_Tcm_ANXA1
CD8_Tnaive_CCR7
FollicularB_MS4A1
CD4_Tnaive_CCR7
FollicularB_IGHD
GC_AICDA
T_cells_proliferative
Monoyte_CX3CR1
Macrophage_MKI67
Telocytes_ICAM1
Monoyte_IL1B
Macrophage_SPP1
iCAF_CXCL12
MyoCAF_ACTA2
Malignant_epithelial_cells_ASCL2
Malignant_epithelial_cells_proliferative
CD8_Tem_GZMK
CD8_Trm_CXCL1
CD4_Th1_CXCL13
NK_cells_KLRB1
CD8_HSPA1A
Endothelial_cell
PVL_MCAM
CD4_Trm_CXCR6
Mast cell
CD4_Treg_FOXP3
Macrophage_SELENOH
Monoyte_NLRP3
CAF_CXCL14
cDC2_CD1C
CD4_CD8_cells
Macrophage_C1QC
DC_LAMP3
Macrophage_FCN1
CD8_SELENOK
CD8_proliferative
CD4_Th17_IL17A
CD8_Tem_FGFBP2
CD8_Tex_PDCD1
NK_cells_KLRD1
CD8_IEL_CD160
CD8_TEMRA_TEFF
CD8_Tem_CD161
NK_cells_FCGR3A
GC_LRMP
Malignant_epithelial_cells_TM4SF1
CAF_FAP
factor 3
factor 2
factor 0
factor 10
factor 7
factor 4
factor 1
factor 6
factor 9
factor 8
factor 5
D
CD4_Tnaive_CCR7
Malignant_epithelial_cells_SOX4
FollicularB_IGHD
CD4_Tcm_ANXA1
FollicularB_MS4A1
CD8_Tnaive_CCR7
Malignant_epithelial_cells_TM4SF1
MyoCAF_ACTA2
Malignant_epithelial_cells_FCGBP
Malignant_epithelial_cells_OLFM4
Macrophage_MKI67
Monoyte_NLRP3
CAF_CXCL14
GC_LRMP
CD4_CD8_cells
CD4_Tem_GZMK
CD4_Th1_CXCL13
CD8_Tem_GZMK
CD8_Trm_CXCL1
CD4_Treg_FOXP3
CD8_Tex_PDCD1
CD8_HSPA1A
CD8_IEL_CD160
NK_cells_KLRD1
Monoyte_CX3CR1
CD8_TEMRA_TEFF
NK_cells_FCGR3A
T05_CD8_Tem_FGFBP2
CD8_Tem_CD161
CD4_Trm_CXCR6
CD4_Th17_IL17A
CD8_SELENOK
PVL_MCAM
Malignant_epithelial_cells_ASCL2
NK_cells_KLRB1
Malignant_epithelial_cells_proliferative
GC_AICDA
Macrophage_FCN1
Macrophage_SELENOH
CD8_proliferative
cDC2_CD1C
DC_LAMP3
Macrophage_C1QC
CAF_FAP
Telocytes_ICAM1
Monoyte_IL1B
T_cells_proliferative
Plasma_IgG
Plasma_IgA
iCAF_CXCL12
Endothelial_cell
Mast cell
Macrophage_SPP1
Expression
1
0.5
0
Percent expressed
0.25
0.5
0.75
1
factor 7
factor 6
factor 1
factor 4
factor 9
factor 10
factor 8
factor 3
factor 0
factor 2
factor 5

Supplementary Figure 8. (A) Spots were assigned with latent factors by NMF (left) or clustered by transcription (middle) in a slice of stage-IV CRC tissue. The image of matched H & E staining was shown at right. **(B)** The clustering of each spot by NMF (left) or transcription (middle) was exhibited in a slice of stage-II CRC tissue. The image of matched H&E staining was illustrated at right. **(C)** Dot plot demonstrated the weight of each cluster contributing to latent factors for ST-seq dataset as same as in (A). **(D)** Dot plot depicted the weight of each cluster contributing to latent factors for ST-seq dataset as same as in (B). NMF; nonnegative matrix factorization; CRC, colorectal cancer; H & E, hematoxylin and eosin; CAF, cancer associated fibroblast; iCAF, inflammatory CAF; PVL, perivascular-like; Trm, memory T cell; Tcm, central memory T cell; Tem, effector memory T cell; TEMRA/TEFF, recently activated effector memory or effector T cell; IEL, intraepithelial lymphocyte; Tex, exhausted T cell; cDC, classical dendritic cell; pDC, plasmacytoid dendritic cell.

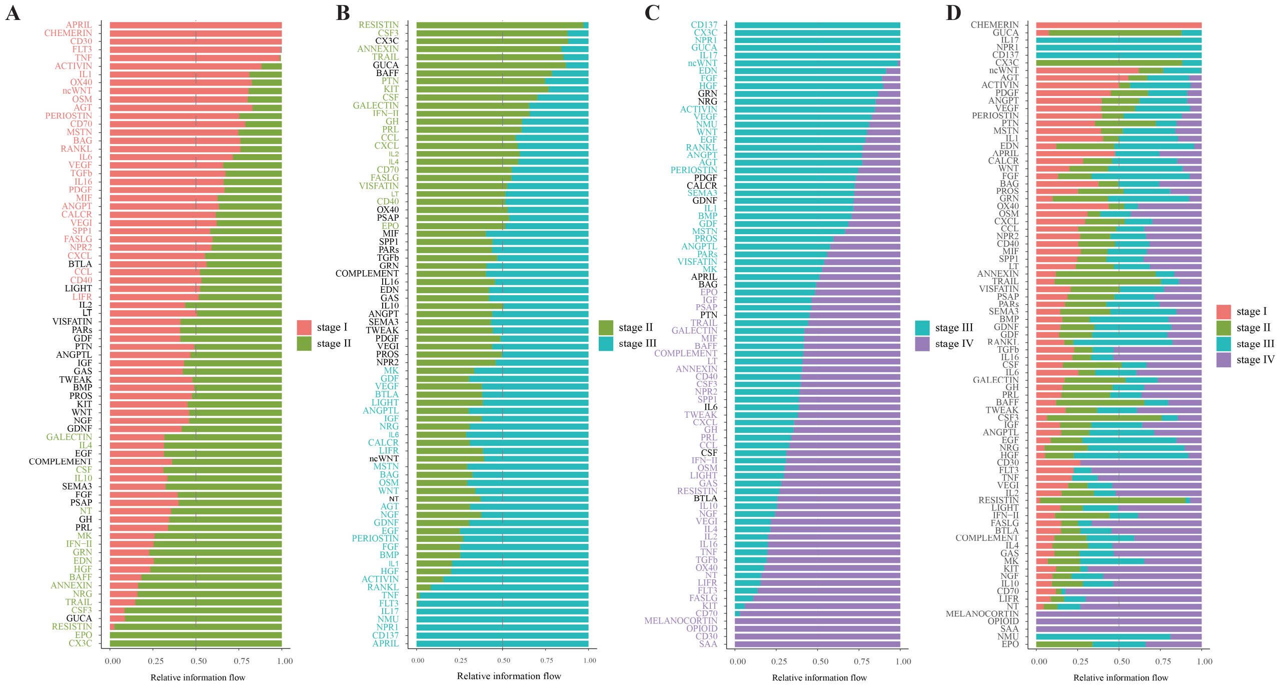

Supplementary Figure 9. (A–D) The relative information flow of enriched pathways by differential expression analysis of ligand-receptors for CRC tissues between stage I and stage II (A), or between stage II and stage III (B), or between stage III and stage IV (C). The distribution of relative information flow of pathways for each stage was show in (D). CRC, colorectal cancer.
